# 4-1BB^+^ Tregs and inhibitory progenitor exhausted T cells confer resistance to anti-PD-L1 and anti-CTLA-4 combination therapy

**DOI:** 10.1016/j.xcrm.2025.102408

**Published:** 2025-10-03

**Authors:** Junha Cha, Chang Gon Kim, Nam Suk Sim, Gamin Kim, Wonrak Son, Dahee Kim, Yurim Jung, Hyun Jun Hong, Hae Been Lee, Jaehyung Kim, Jinna Kim, Sun Och Yoon, Seokhyeong Go, Jeongah Kim, Euijung Seong, Seungbyn Baek, Kyung Hwan Kim, Min Hee Hong, Yoon Woo Koh, Insuk Lee, Hye Ryun Kim

**Affiliations:** 1 Department of Biotechnology, College of Life Science and Biotechnology, Yonsei University, Seoul 03722, Republic of Korea; 2 Division of Oncology, Department of Medicine, Stanford University School of Medicine, Stanford, CA 94305, USA; 3 Division of Medical Oncology, Department of Internal Medicine, Yonsei Cancer Center, Yonsei University College of Medicine, Seoul 03722, Republic of Korea; 4 Department of Otorhinolaryngology, Yonsei University College of Medicine, Seoul 03722, Republic of Korea; 5 Department of Radiology, Yonsei University College of Medicine, Seoul 03722, Republic of Korea; 6 Department of Pathology, Yonsei University College of Medicine, Severance Hospital, Seoul 03722, Republic of Korea; 7 Department of Radiation Oncology, Yonsei Cancer Center, Heavy Ion Therapy Research Institute, Yonsei University College of Medicine, Seoul 03722, Republic of Korea; 8 POSTECH Biotech Center, Pohang University of Science and Technology (POSTECH), Pohang 37673, Republic of Korea; 9 Division of Medical Oncology, Department of Internal Medicine, Yonsei Cancer Center, Graduate School of Medical Science, Brain Korea 21 Project, Yonsei University College of Medicine, Seoul 03722, Republic of Korea

**Keywords:** immunotherapy, immune checkpoint inhibitor, dual ICB, durvalumab, tremelimumab, head and neck squamous cell carcinoma, progenitor exhausted T, single-cell RNA, single-cell TCR

## Abstract

Predictors of immune checkpoint inhibitor response in cancer remain elusive. From a previous phase 2 neoadjuvant immunotherapy window-of-opportunity study, we present the single-cell RNA and T cell receptor (TCR) sequencing analysis of 57 pre- and post-treatment tumor biopsies from head and neck cancer patients treated with durvalumab (anti-PD-L1) alone or with tremelimumab (anti-CTLA-4), identifying key cellular and molecular predictors of immune checkpoint inhibitor (ICI) response. Malignant cells and neutrophil senescence promote ICI response. While *CXCL13*^*+*^ exhausted T (Tex) cells enhance response through 4-1BB signaling, anti-CTLA-4 induces 4-1BB^+^ regulatory T cells (Tregs) restricting ICI efficacy. These opposing roles of 4-1BB in different cellular contexts may explain the limited benefit of combinatorial immunotherapy observed in clinical trials. We identify two subsets of tumor-reactive progenitor Tex (Tpex): ICI-responsive Tpex1 and ICI-resistant Tpex2, a subset characterized by *KLRB1* and *IL17R*. The balance of Tpex1 and Tpex2 associates with ICI response across multiple cancers, offering insights into sustaining response. This study was registered at ClinicalTrials.gov (NCT03737968).

## Highlights

• Addition of anti-CTLA-4 to anti-PD-L1 promotes 4-1BB^+^ Tregs, restricting ICI response
• Two progenitor exhausted T cells exist: ICI-responsive Tpex1 and ICI-resistant Tpex2
• The balance of Tpex1, Tpex2, and Tex is associated with ICI response across cancers
• Senescence of malignant cell and neutrophil promotes ICI response

## Graphical abstract

**Figure:**
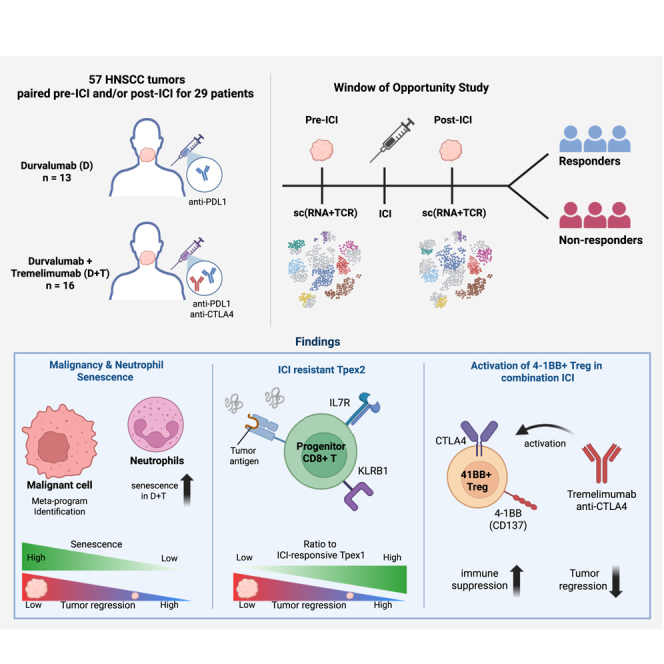

## Teaser

Cha et al. show existence of immunotherapy-resistant progenitor exhausted T cells, demonstrating a key cellular factor determining successful cancer immunotherapy. Conflicting mechanisms both suppressing and promoting tumor are associated with anti-CTLA-4 and explain their varied clinical results.

## Introduction

Head and neck cancer is a type of malignancy, with squamous cell carcinoma accounting for over 90% of cases.^1^ Recently approved immune checkpoint inhibitors (ICIs) such as nivolumab and pembrolizumab have changed treatment plan for recurrent and metastatic head and neck squamous carcinoma (R/M HNSCC). Based on the success of ICI in improving overall survival in R/M HNSCC, various clinical trials are currently underway to investigate the potential safety and effect of ICIs in patients with early-stage HNSCC (e.g., NCT03765918).^2^ However, despite much effort to find the biomarker predicting ICI response, validated biomarkers for immunotherapy have remained elusive, and it is also unclear which components of the tumor microenvironment (TME) dictate treatment outcomes of ICIs. Moreover, while it has been well documented that human papilloma virus (HPV) infection status of the patients with oropharyngeal cancer is a significant predictor of survival,^3^ its association with ICI response has not been determined.

Combination immunotherapy targeting multiple immune checkpoints such as CTLA-4 and PD-(L)1, has been expected to increase therapeutic efficacy,^4^ given that the majority of patients with head and neck cancer either fail to respond or relapse with PD-(L)1 blockade as a single agent.^5^^,^^6^ This hypothesis was evaluated in CheckMate 651 (NCT02741570)^7^ and KESTREL study (NCT02551159),^8^ demonstrating that combined checkpoint blockade of PD-(L)1 and CTLA-4 has shown clinical benefit in some subgroups of patients rather than overall populations. Hence, it is important to understand cellular and molecular mechanisms of PD-(L)1 and CTLA-4 blockade either as a monotherapy or combination to design effective treatment options for individual patients.

Window-of-opportunity studies exploit the period between cancer diagnosis and definitive treatment, to explore novel therapeutic strategies. In this study, we analyzed the TMEs of pre- and post-treatment tumor specimens to identify molecular and cellular determinants of early ICI response and resistance. Patients received neoadjuvant durvalumab (D, anti-PD-L1 antibody) alone or in combination with tremelimumab (T, anti-CTLA-4 antibody) in a prospective randomized phase 2 trial (NCT03737968).^9^ We employed integrative RNA and T cell receptor (TCR) profiling at the single-cell level, correlating TME characteristics with pathologic tumor regression. Through this approach, we illustrate key aspects of the tumor immune ecosystem and provide insights into the mechanisms underlying neoadjuvant ICI response and resistance in HNSCC.

We employed various computational approaches for single-cell data analysis to explore the TME in ICI-treated patients with HNSCC. To address cancer heterogeneity in assessing malignant gene programs, non-negative matrix factorization (NMF) approach has been applied to single-cell data analysis.^10^^,^^11^ In addition, foundation models trained on millions of single cells,^12^ analogous to large language models, offer a powerful framework for studying gene and cell functions in the TME. By re-training these models on ICI response data, we can identify key genes shaping their embeddings in the latent space. A network-based approach^13^ to tumor-experienced CD8^+^ T cells revealed central genes that showed functional difference across ICI responses. Furthermore, RNA velocity of tumor-reactive T cells,^14^ identified through clonotype sharing with exhausted subsets,^15^ revealed the differential potential of progenitor exhausted T cells present in baseline tumors. This population appears to be highly associated with neoadjuvant ICI response.^16^

## Results

### Window-of-opportunity study design for HNSCC neoadjuvant ICI clinical trial

**Figure 1:**
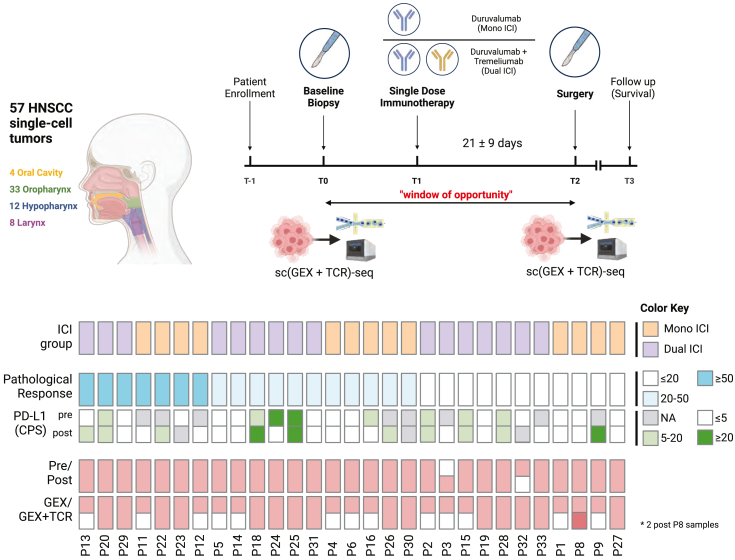
Overall research design of HNSCC immunotherapy clinical trial Study design and sampling scheme of the window-of-opportunity study. Study timeline for enrolled samples and their description of treatment, pathological response, and the generated data type are illustrated.

Patients diagnosed with HNSCC who underwent surgical resection at Severance Hospital (Seoul, Republic of Korea) between January 2019 and December 2020 were enrolled in a previously reported prospective randomized trial (NCT03737968, Figure 1). Clinical metadata are summarized in Table S1. We analyzed a total of 29 HNSCC patients with available single-cell transcriptomic data. Patients received a single cycle of either intravenous durvalumab (D, 1,500 mg; *n* = 13) or a combination of durvalumab and tremelimumab (D + T, 1,500 mg + 75 mg; *n* = 16), followed by curative surgery performed 2 to 8 weeks after ICI administration. Computed tomography or magnetic resonance imaging were performed at baseline and before the surgery. All surgeries were conducted based on the original clinical and radiological extent of the disease. Adjuvant chemoradiotherapy was determined by a multidisciplinary team. Clinical and radiologic follow-up were performed every 3 months during the first 2 years and then every 6 months thereafter. No dose adjustments were permitted for either neoadjuvant D ± T or adjuvant D treatment. A patient with a pathological tumor regression value of ≥50% was defined as an ICI responder^16^^,^^17^^,^^18^^,^^19^; otherwise, the patient was classified as a non-responder.

We collected 28 pre-treatment tumor biopsies and 29 post-treatment tumor samples from surgery, including 27 paired samples. The 57 tumor samples originated from various regions: oral cavities (*n* = 4), oropharynx (*n* = 33), hypopharynx (*n* = 12), and larynx (*n* = 8). To investigate the cellular and molecular mechanisms of ICI responses within tumor tissue, we generated single-cell RNA sequencing (scRNA-seq) and single-cell TCR sequencing (scTCR-seq) data using the 10× Chromium Next GEM (5′ v.2) platform.

### The senescence program of malignant cells is associated with early ICI responses

**Figure 2:**
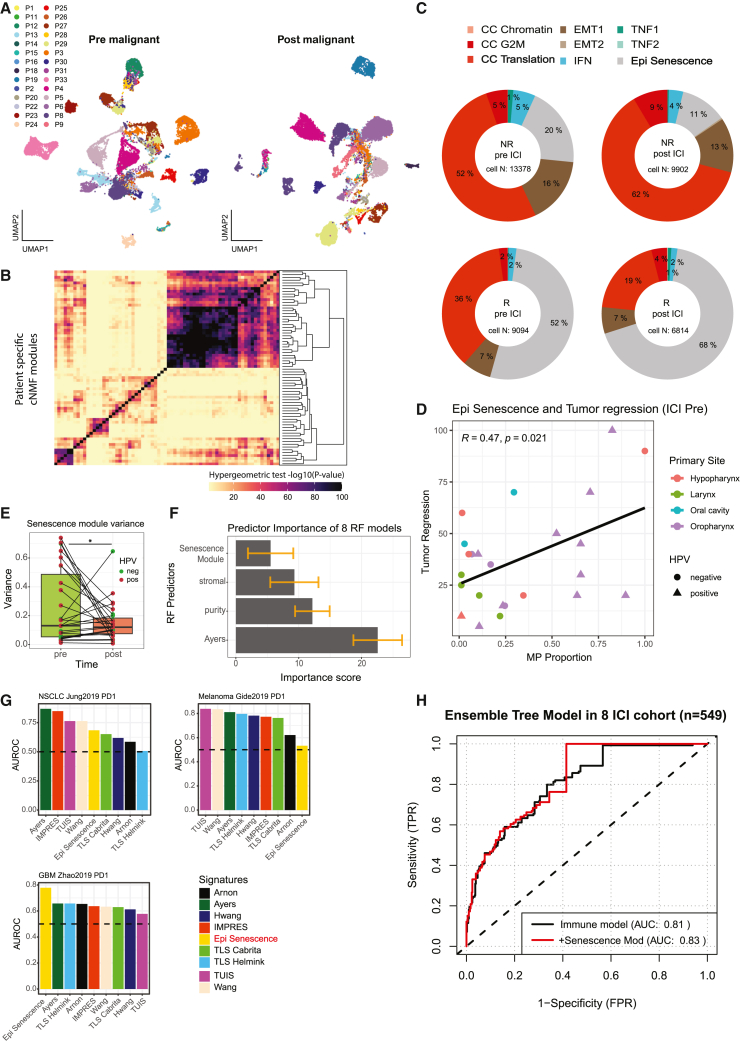
Malignant senescence program associated with ICI sensitivity (A) Uniform manifold approximation and projection (UMAP) of identified malignant cells pre-treatment (left) and post-treatment (right) colored by patient origin. (B) Overlap of selected patient-specific NMF modules. Rows and column clustered with hierarchical clustering. Color indicates −log10 of *p* value calculated via one-sided hypergeometric test (overlap of gene sets). (C) Pie chart proportion of nine meta-programs in pre- and post-ICI responders and non-responder malignant cells. Each cell is labeled a meta-program with the highest gene set module score of the nine. (D) Scatterplot of arithmetic mean of epithelial senescence meta-program score for each pre-ICI patient sample and their tumor regression values post-ICI. Pearson correlation coefficient and its *p* value are denoted. (E) Variance of senescence module score for malignant cells in pre- or post-ICI samples. *p* value is calculated with Levene’s test. (F) Variable importance score for each predictor (calculated as the mean decrease of Gini impurity when a variable is chosen to split a node) of 8 random forest models. Data are represented as mean ± SEM. (G) Area under reciever operating characteristic (AUROC) curve for public signatures in NSCLC dataset, melanoma dataset, and GBM dataset. Dashed line indicates random expectation value of 0.5. Gene signature scores are calculated via gene set variation analysis (GSVA) for each sample. (H) ROC curve for ensemble random forest model without senescence module as a predictor (black, immune model) and with it (red). For both models, tumor purity, stromal score, and immune signature (Ayer et al.^21^) were used as predictors. FPR, false positive rate; TPR, true positive rate

We first aimed to identify gene expression programs of malignant cells associated with ICI response in HNSCC patients by analyzing single-cell gene expression data from 57 tumor tissues. Malignant cells were identified using marker genes and the reference-based classification tool scATOMIC^20^ (STAR Methods). To overcome transcriptional heterogeneity of malignant cells (Figures 2A and S1A), a non-negative factorization (NMF) method was applied to individual patients to retrieve gene programs found across the entire sample cohort (Figure S2A, STAR Methods). No specific copy-number variation was associated with patient-level phenotypes (Figure S2B). While these factorized gene sets exhibited high patient specificity as expected, we also found gene sets that significantly overlapped across patients (Figure 2B). We clustered these gene sets into nine non-overlapping, functionally distinct meta-programs (Figure S1B) and assessed their representative functions through term enrichment and manual curation compared to previously established tumor meta-programs.^10^^,^^11^ Functional annotations of the identified nine meta-programs and their member genes are summarized in Table S2.

We assumed that each individual cell exhibits a dominant meta-program driving carcinogenesis. By analyzing the proportion of the highest-scored representative meta-programs per patient, we observed a negative correlation between the “CC translation” meta-program and tumor regression, whereas the “epithelial senescence” meta-program showed a positive correlation (Figure S1C). Patients with higher tumor regression, indicative of a better ICI response, tended to have malignant cells with lower CC Translation and higher epithelial senescence meta-program scores. Notably, epithelial senescence is associated with reduced stemness, potentially leading to less aggressive tumor in HNSCC.^22^^,^^23^ Additionally, we found that the epithelial senescence meta-program was enriched for immune functions (Figures S1D and S1E). When comparing groups by treatment time and response, we observed that these two meta-programs drove HNSCC malignancy and were clearly distinguished in proportion based on ICI response (Figure 2C). Importantly, the epithelial senescence program could predict ICI response at baseline, with HPV-positive oropharynx samples displaying notable high senescence program (Figure 2D). Within the HPV-positive oropharyngeal cancer, the epithelial senescence program exhibited a higher proportion both at baseline and post-ICI treatment, in higher tumor regression sample compared to low (Figure S2C).

To further investigate the core module of the epithelial senescence program for ICI response, we dissected the meta-programs using the gene co-occurrence network of NMFs (STAR Methods). This approach enabled us to identify 28 core genes within the epithelial senescence meta-program, which we refer to as the “senescence module” (Table S3). The expression variance of this module significantly decreases post-treatment (Figure 2E), indicating that malignant cells with high senescence module activity are sensitive to ICIs. Additionally, the senescence module score showed a significant association with tumor regression (Figure S1F). Using publicly available tumor bulk RNA sequencing (RNA-seq) data with response evaluation criteria in solid tumors (RECIST) annotations, we found that incorporating the senescence module score as a separate feature in the random forest classifier improved the prediction of ICI response (STAR Methods). We found that, while the representative immune signature,^21^ tumor purity, and stromal score^24^ were important predictors, the senescence module score demonstrated notable importance across most cohorts, despite the convoluted expression of tumor biopsies (Figure 2F). In canonical “hot tumors” such as non-small cell lung cancer (NSCLC) and melanoma, public immune signatures^21^^,^^25^^,^^26^^,^^27^^,^^28^^,^^29^^,^^30^^,^^31^ performed well in retrieving responders (Figure 2G). However, in the cold tumor glioblastoma (GBM), the senescence module outperformed other immune signatures in prediction accuracy. When we integrated eight random forest classifiers from each cohort as an ensemble model (STAR Methods), the addition of the senescence module scores improved response prediction for samples, especially where response and non-response distinctions are not clear (0.4 < FPR < 0.6, Figure 2H). These findings suggest that gene expression programs within malignant cells of the TME can influence ICI responses.

### *CXCL13*^*+*^ exhausted T cells and their 4-1BB signaling are linked to early ICI response

**Figure 3:**
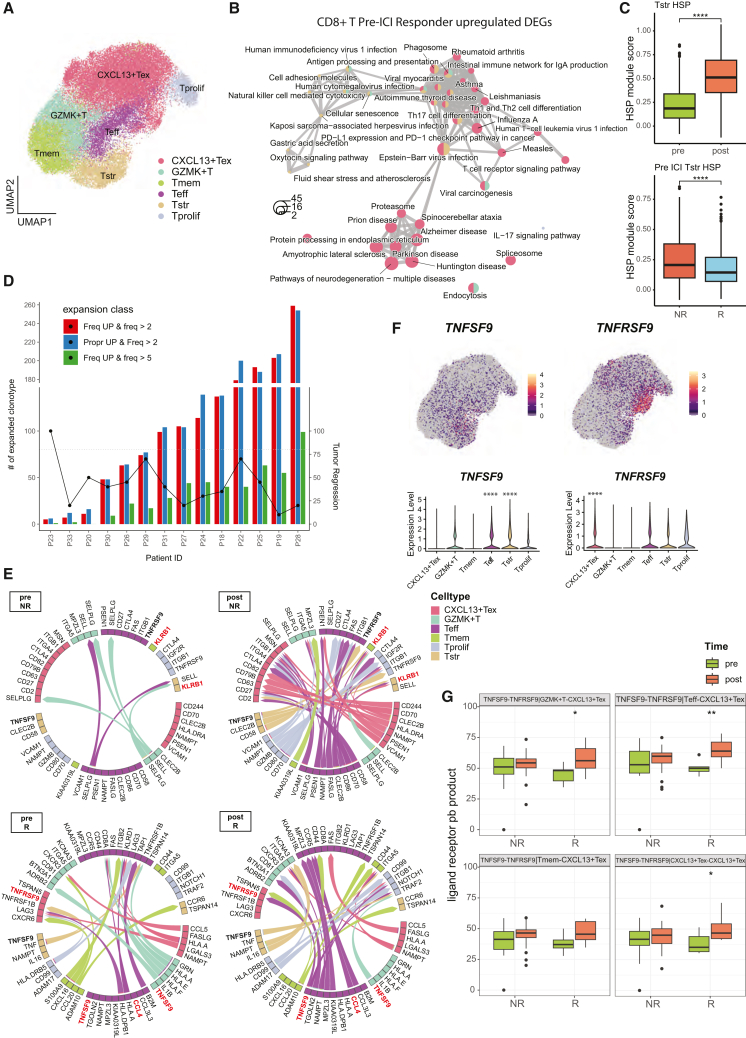
ICI response predictors in CD8^+^ T states (A) UMAP of CD8^+^ T cells extracted, re-normalized, and re-clustered. Tex, exhausted T cell; Tmem, memory T cell; Teff, effector T cell; Tstr, stressed T cell; Tprolif, proliferating T cell. (B) Network representation of enriched KEGG pathway for pre-ICI CD8^+^ T cells upregulated in responders (R) compared to non-responders (NR), visualized via *emapplot()* of clusterProfiler package. Colors indicate the enriched subcluster within CD8^+^ T cells. Size represents the overlapping genes in each term. Color within the term node represents the contribution of that cluster to the term based on the number of overlapping genes from each cluster. (C) Boxplot of stress-associated heat shock protein (HSP) signature identified to be upregulated in Tstr, significantly less so in responders at baseline. *p* values were calculated via Wilcoxon rank-sum test. (D) The number of expanded T cell clonotypes in post- versus pre-treatment tumor biopsies, classified by expansion of frequency (above 2 red and above 5 green) and expansion of proportion and frequency (blue). Overlaid scatterplot indicates tumor regression value post-therapy. (E) Top 50 intracellular ligand-receptor pairs across CD8^+^ T subclusters that significantly increased interaction post-therapy for each response group as measured by MultiNicheNet. *TNFSF9* and *KLRB1* are highlighted in red. (F) Feature plot of *TNFSF9* expression in CD8^+^ T cell UMAP dimension and violin plot of *TNFSF9* expression for each subclusters (left) and feature plot of *TNFRSF9* expression in CD8^+^ T cell UMAP dimension and violin plot of *TNFRSF9* expression for each subclusters (right). *p* values were calculated via Wilcoxon rank-sum test for each cluster vs. all others, adjusted by Benjamini-Hochberg method. (G) Cell-type-specific ligand-receptor pseudo-bulk (by patient) product value from MultiNicheNet of top 8^th^ to 11^th^ that showed statistically significant increase in responder vs. non-responders post-therapy compared to baseline. Two-sided *t* test was additionally performed and denoted at the top of each group comparison (pre- vs. post-ICI). *p* value significance: ∗*p* < 0.05, ∗∗*p* < 0.01, ∗∗∗*p* < 0.001, and ∗∗∗∗*p* < 0.0001.

Pre-existing resident memory CD8^+^ T cells have been shown to play critical role in neoadjuvant immunotherapy.^16^ To investigate immune mechanisms predicting ICI response, we focused on CD8^+^ T cell subpopulations among all identified immune cells (Figure S3A). We subclustered CD8^+^ T cells into six subtypes (Figure 3A) and identified differentially upregulated genes across these subtypes (Table S4). Gene set enrichment analysis of differentially expressed genes (DEGs) upregulated in responders compared to non-responders at baseline revealed significant enrichment of Kyoto Encyclopedia of Genes and Genomes (KEGG) pathways related to immunity and immune disorders. Notably, genes upregulated in *CXCL13*^+^ exhausted T cells (*CXCL13*^*+*^ Tex) were enriched in responder-associated pathways such as PD-L1 expression and PD-1 checkpoint pathway in cancer and TCR signaling pathway for CD8^+^ T cell subtypes (Figure 3B). *CXCL13*^*+*^ Tex cells expressed tumor-experienced markers such as *ENTPD1* and *ITGAE*,^32^ cytotoxic genes (*PRF1*, *GZMA*, and *IFNG*), and exhaustion markers such as *PDCD1* (Figure S4). These findings suggest that *CXCL13*^*+*^ Tex cells are a key subset contributing to ICI response.

Previous studies have reported that stress-related genes are strongly associated with ICI resistance.^33^ Consistent with this, we extracted the top expressed genes from the stressed T cell (Tstr) subcluster (Figure S3B) and evaluated their gene set enrichment scores across different groups (STAR methods). Signature genes for this cell state indicative of ICI resistance^33^ were upregulated post-ICI treatment and significantly distinguished responders from non-responders at baseline and treatment group post-therapy (Figures 3C and S3C). Independent differential expression analysis using a negative binomial mixed model (NEBULA)^34^ with patient heterogeneity as a random effect (STAR Methods) confirmed that stress-associated genes were associated with ICI administration and response (Table S5).

Neoadjuvant ICI response is challenging to assess using conventional radiologic RECIST criteria. Instead, post-treatment TCR clonal expansion compared to baseline is often used as a surrogate response metric.^35^ In our HNSCC cohort, most patients showed significant clonal expansion after a single treatment cycle (Figure S5A). However, TCR expansion did not correlate with neoadjuvant tumor regression (Figure 3D) in any CD8^+^ T cell subtypes, regardless of whether patients received monotherapy or combination therapy (Figure S5B). Clonal expansion was predominantly restricted to the *CXCL13*^*+*^ Tex subset (Figure S3D), confirming this subtype as tumor reactive. Analysis of TCR clonotypes that emerged, persisted, or were lost after ICI treatment revealed that persisting clonotypes were highly expanded, but no significant differences were observed between response groups or treatment arms (Figure S3E).

Cell-cell interaction (CCI) analysis among CD8^+^ T cell subtypes revealed key genes contributing to ICI response. Top intercellular ligand-receptor interactions, deconvolved for patient heterogeneity (STAR Methods), showed notable differences between response groups (Figure 3E). In the non-responders, interactions with *KLRB1* (encoding CD161) in memory T cell (Tmem) and Tstr increased post-ICI treatment, supporting ICI resistance. This aligns with our previous finding that CD161^+^ tissue-resident memory T cells counteract clinical benefits in HPV-infected patients.^36^ In contrast, responders showed increased interactions involving *TNFRSF9* (encoding 4-1BB) in *CXCL13*^*+*^ Tex after ICI treatment, suggesting a positive role in ICI response. *TNFRSF9* and its ligand *TNFSF9* were predominantly expressed in effector T cell (Teff), Tstr, and *CXCL13*^*+*^ Tex (Figure 3F). Differential ligand-receptor activity analysis in *CXCL13*^*+*^ Tex using MultiNicheNet (https://github.com/saeyslab/multinichenetr) revealed that *TNFSF9*-*TNFRSF9* activity increased more significantly in responders following ICI treatment (Figure 3G). The differential magnitude of the TNFSF9-TNFRSF9 interaction was further validated using a dual immunohistochemistry assay (Figure S6, STAR Methods). These findings suggest that 4-1BB signaling in *CXCL13*^*+*^ Tex contributes positively to early ICI response.

### Network and foundation models reveal key *CXCL13*^*+*^ Tex genes for early ICI response

**Figure 4:**
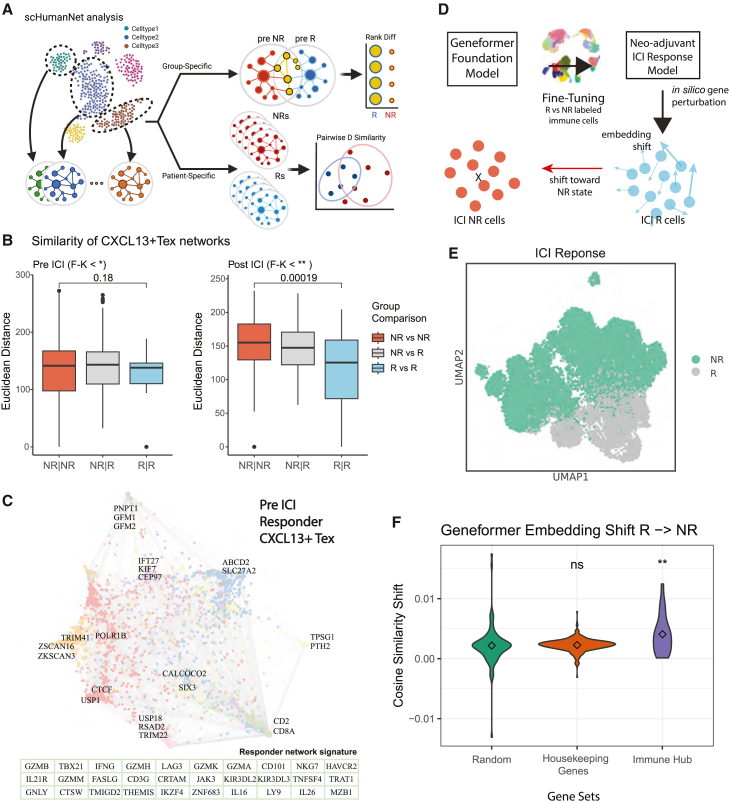
*CXCL13*^*+*^ Tex network topology as a predictor for ICI response (A) Conceptual depiction of the network analysis using scHumanNet performed for *CXCL13*^*+*^ Tex cells. (B) Similarity of patient-specific network nodes calculated with pairwise Euclidean distance from adjacency matrix of a union gene set. *p* values were calculated via two-sided *t* test. Additionally, difference of variance was tested with Fligner-Killeen (F-K) test, and its *p* values are denoted. (C) Cell-type-specific network of responder *CXCL13*^*+*^ Tex from pre-ICI treatment group. Gene nodes are colored according to each different subcommunity determined by Louvain clustering with top 3 nodes depicted for each community. The 30 genes ordered by centrality in a responder community that decreased in centrality in the corresponding non-responder network are summarized as a table below (green) and termed responder network signature. (D) Conceptual depiction of the *in silico* perturbation analysis using a fine-tuned (all immune cells) Geneformer foundation model. For each gene perturbed, the embedding shift of responder cells (*CXCL13*^*+*^ Tex only) toward non-responder cells was measured through cosine similarity. (E) Two-dimensional UMAP representation of fine-tuned Geneformer embeddings (512 dimensions) with immune cells as input, labeled for ICI response status. (F) Cosine similarity of shifted embedding for each gene set. 100 random genes were sampled from perturbed genes as control. The 21 genes of the immune hub signature show positive shift toward non-response when perturbed. *p* value significance: ∗*p* < 0.05, ∗∗*p* < 0.01, ∗∗∗*p* < 0.001, and ∗∗∗∗*p* < 0.0001; ns, non-significant.

Single-cell gene expression data enable the construction of cell-type- or patient-specific gene networks, with topological analysis complementing expression-based research.^37^^,^^38^ To explore gene regulatory mechanisms in *CXCL13*^*+*^ Tex for early ICI response, we built *CXCL13*^*+*^ Tex gene networks for two response groups and individual patients (Figure 4A, STAR Methods). Responder-specific gene networks showed significantly higher node-profile similarity compared to non-responders, particularly post-ICI treatment (Figure 4B). This pattern aligns with the Anna Karenina principle in microbiome research,^39^ where dysbiotic individuals exhibit more variability in taxonomic profiles compared to healthy individuals due to the presence of core taxa in healthy microbiomes. Similarly, *CXCL13*^*+*^ Tex gene networks in responders shared more common nodes, suggesting the existence of core regulatory genes driving ICI response. Supporting this, T cell gene networks from adjacent normal tissues in lung and colorectal cancer patients showed greater node-profile similarity compared to networks from tumor-infiltrating T cells (Figure S3F), indicating shared regulatory structures in healthy cellular contexts.^40^

To define genes that characterize *CXCL13*^*+*^ Tex driving ICI response, we identified the top 30 differential hub genes in the responder *CXCL13*^*+*^ Tex network at baseline compared to the non-responder network, referred to as the “responder network signature” (Figures 4C; Table S6). This signature includes key genes for maintaining cellular cytotoxicity such as *ZNF683*,^41^ *PRF1*, *IL2RB*, and *IFNG* and immune activation and exhaustion markers including *LAG3*, *PDCD1*, *PRF1*, and *TBX21* that contribute to anti-tumor immunity.

To validate the role of responder network signature genes in maintaining the regulatory network, we simulated network state alterations between responders and non-responders using “virtual knockout” scheme from the Geneformer foundation model,^12^ pre-trained on atlas-scale single-cell transcriptome data. We fine-tuned the Geneformer model with our immune cell scRNA-seq data, creating a *CXCL13*^*+*^ Tex-specific model where 24 out of 30 responder signature genes remained. *In silico* gene perturbation of these 24 genes was performed to assess whether responder *CXCL13*^*+*^ Tex cells shifted toward a non-responder embedding profile (Figure 4D). The fine-tuned model efficiently distinguished immune cells of responders from non-responders (Figure 4E, STAR Methods). Compared to random or housekeeping genes, perturbation of 19 out of 24 responder signature genes significantly shifted responder *CXCL13*^*+*^ Tex cells toward a non-responder profile (Figures 4F; Table S7). This finding suggests that the responder network signature genes of *CXCL13*^*+*^ Tex cells play key regulatory roles in promoting ICI response.

### An inhibitory progenitor Tex subset confers ICI resistance

**Figure 5:**
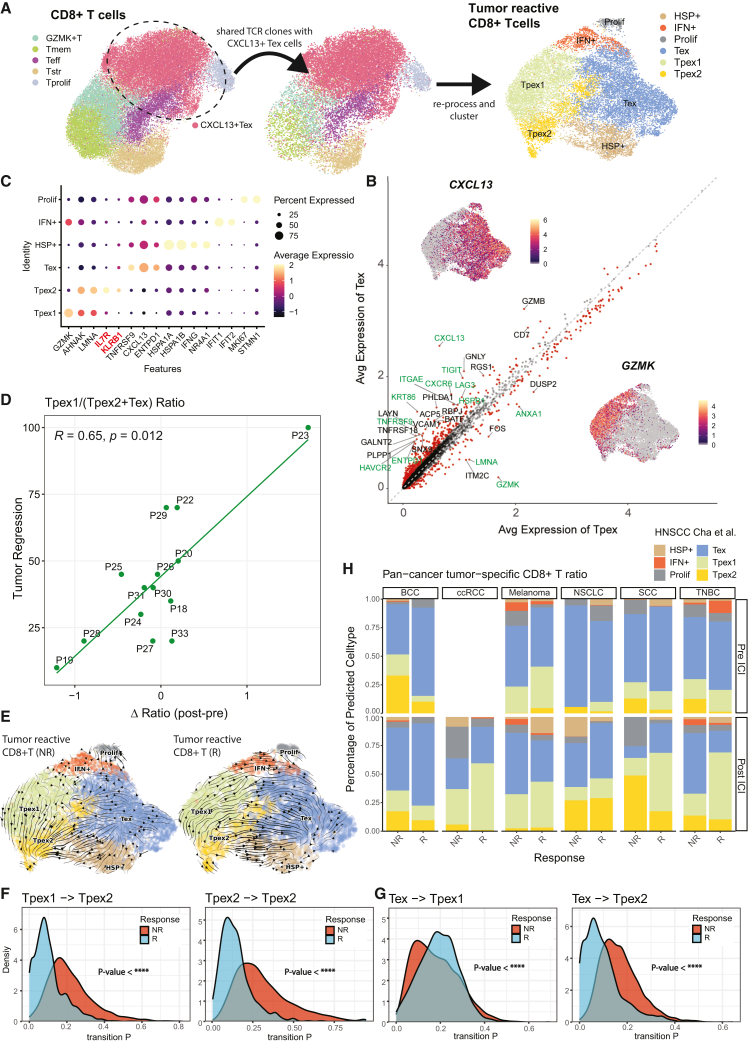
Identification of ICI-resistant Tpex subpopulation (A) Conceptual depiction of TCR-based selection and identification of tumor-specific exhausted T cells (Tex) and progenitor exhausted T cells (Tpex). (B) Dot plot of selected genes significantly upregulated in specific subsets of tumor-specific CD8^+^ T cells. (C) Average expression genes in Tex and Tpex (Tpex1 + Tpex2). Red dots indicate significantly upregulated genes in each group calculated by two-sided *t* test. Key genes associated with memory/exhaustion/dysfunction are highlighted in green. (D) Scatterplot of ratio differences (post-ICI minus pre-ICI) for patients with TCR information. Pearson correlation coefficient and its *p* value is depicted. Ratio were calculated as Tpex1/(Tpex2 + Tex). (E) RNA velocity streamline plot for tumor-reactive CD8^+^ T cells in non-responder group (left) and responder group (right). (F) Cell transition probability density for Tpex1 to Tpex2 (left) and persistence of Tpex2 (right). (G) Reverse transition of Tex to Tpex1 (left) and Tpex2 (right), for non-responder and responder group. *p* values are calculated via Kolmogorov-Smirnov test. (H) Proportion of tumor-specific CD8^+^ T subclusters identified via Seurat label transfer algorithm. Datasets were divided by cancer type, ICI treatment, and response group as annotated by the original authors. *p* value significance: ∗*p* < 0.05, ∗∗*p* < 0.01, ∗∗∗*p* < 0.001, and ∗∗∗∗*p* < 0.0001; ns, non-significant.

Not all tumor-infiltrating CD8^+^ T cells are tumor antigen specific,^42^ and conventional expression-based clustering fails to distinguish tumor-reactive subsets. Clonotype analysis enables precise identification of tumor-specific T cells including progenitor Tex (Tpex).^15^ We analyzed clonotypes within the *CXCL13*^+^ Tex subset and filtered for subpopulations sharing these clonotypes (STAR Methods). Subclustering analysis for tumor-reactive CD8^+^ T cells identified Tpex subsets (Tpex1 and Tpex2) and Tex (Figures 5A and S7A; Table S8). Tpex subsets were characterized by high *GZMK* expression and memory-associated genes (*LMNA* and *AHNAK*), while Tex exhibited high expression of *CXCL13*, residency markers (*ITGAE*, *ENTPD1*, and *CXCR6*), immune checkpoints (*TIGIT*, *LAG3*, and *HAVCR2*), dysfunction-associated genes (*KRT86* and *HSPB1*), and *TNFRSF9* (Figures 5B, 5C, and S8).

Tpex1 corresponds to the previously reported Tpex, a key determinant of successful immunotherapy.^43^^,^^44^ We identified a subset, Tpex2, distinguished from Tpex1 by *KLRB1* and *IL7R* co-expression, which may play a role in establishing tumor-specific memory^45^ (Figures 5C and S7B). Consistent with previous reports, Tpex1 was enriched in the tumor immune microenvironment of early responders, while Tex and Tpex2 proportions decreased (Figure S7C). Additionally, changes in the Tpex1-to-(Tex + Tpex2) ratio between baseline and post-ICI treatment correlated with tumor regression (Figure 5D). These findings suggest that Tpex2 may inhibit ICI response.

Trajectory analysis identified three differentiation lineages from Tpex1 (Figure S7D), two of which led to dysfunctional tumor-reactive CD8^+^ T cells with Tpex2 as an intermediate: Tpex1 → Tpex2 → Tex (lineage 1, Figure S7E) and Tpex1 → Tpex2 → HSP^+^ cells (lineage 2, Figure S7F). Responders were enriched in Tpex1, whereas non-responders had higher proportions of Tpex2, Tex, and HSP^+^ cells (Figures S7G and S7H). Along lineage 1, *CXCR5*, *SOX4*, *CCR7*, and *BATF* showed differential expression dynamics between responders and non-responders (Figure S7I, STAR Methods), while ICI resistance genes such as *KRT86*^46^ and *HSPB1* were upregulated along lineage 2 in non-responders (Figure S7J). Key genes whose expression was significantly associated with pseudotime (adjusted *p* value < 0.05) included previously identified Tpex markers such as *TCF7* (Figure S9A; Table S9). Gene ontology enrichment analysis revealed that these genes are involved in T cell activation and CD8^+^ T cell-mediated anti-tumor responses (Figure S9B). RNA velocity analysis indicated differential cellular transition toward Tpex2 in tumor-reactive CD8^+^ T cells across ICI response group (Figure 5E). Cell-to-cell transition probability analysis (STAR Methods) further revealed increased Tpex1-to-Tpex2 transition and greater Tpex2 persistence in non-responders (Figure 5F). Tex exhibited distinct transition probabilities toward Tpex1 and Tpex2, with a higher likelihood of transitioning to Tpex1 and a lower likelihood to Tpex2 in responders, underscoring their opposing roles in ICI response (Figure 5G). Consistent with prior reports on Tpex stemness during ICI therapy,^47^ we observed increased stemness in both Tpex1 and Tpex2 post-treatment compared to other subsets (Figure S7K), suggesting their contribution to early ICI response.

Overall, our results suggest that Tpex2 may confer ICI resistance during human neoadjuvant immunotherapy. To assess whether Tpex2 is resistant to ICI response across cancer types beyond HNSCC, we analyzed single-cell transcriptome data from ICI-treated samples of various cancers and compared tumor-reactive CD8^+^ T cell subsets using a label transfer approach (STAR Methods). Notably, ICI-resistant Tpex2 subsets were more abundant in non-responders in both pre- and post-treated samples across multiple cancer types (Figure 5H), suggesting that Tpex2-mediated ICI resistance may be generalized to diverse cancer types.

### Addition of anti-CTLA-4 to anti-PD-L1 promotes 4-1BB^+^ Tregs restricting ICI response

**Figure 6:**
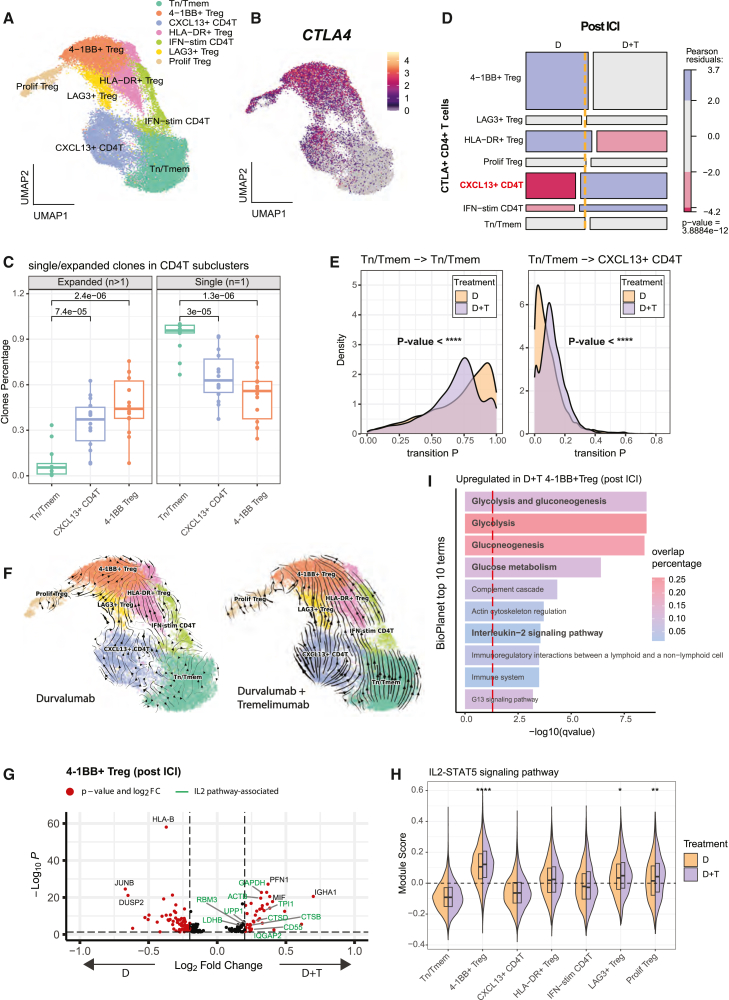
Combinatorial ICI effect inhibition in the CD4^+^ T compartments (A) UMAP of CD4^+^ T cells extracted, re-normalized, and re-clustered. (B) *CTLA-4* expression colored in the CD4^+^ T cell UMAP dimension. (C) Percentage of clones that are expanded (*n* > 1) and that are single (*n* = 1) for three CD4^+^ T cell types. *p* values are calculated via Wilcoxon rank-sum test. (D) Mosaic plot of post-ICI CD4^+^ T cells that express *CTLA-4* (expression above 0). The yellow line indicates the expected ratio. Pearson residual *p* values are colored red or blue if significantly depleted or enriched, respectively. (E) Transition probability density of selected cell-to-cell transitions for monotherapy (D) and combination group (D + T). *p* values are calculated via Kolmogorov-Smirnov test. (F) RNA velocity streamline plot for CD4^+^ T cells in monotherapy group (left) and combination group (right). (G) Volcano plot of differentially expressed genes in 4-1BB^+^ Tregs with positive log fold values for combination group. Genes above 0.25 log2 fold change and under adjusted *p* value (Benjamini-Hochberg) of 0.05 are colored in red. Green text indicates genes associated with IL-2 signaling. (H) Split violin plot for module score of “IL-2-STAT5 signaling” term (collected from BioPlanet 2024) in CD4^+^ T cell subclusters divided by therapy group. (I) Top 10 enriched terms (from BioPlanet database) of 4-1BB^+^ upregulated DEGs in combination group, sorted by *q* value, adjusted via Benjamini-Hochberg method. The red dashed line indicates adjusted *p* value of 0.05 *p* value significance: ∗*p* < 0.05, ∗∗*p* < 0.01, ∗∗∗*p* < 0.001, and ∗∗∗∗*p* < 0.0001; ns, non-significant.

While a previous study showed that addition of anti-CTLA-4 to anti-PD-L1 enhanced activation of CD4^+^ T cells in HNSCC,^48^ this combination unexpectedly led to the expansion of tumor-associated regulatory T cells (Tregs) due to disruption of a CTLA-4-dependent feedback loop, which may limit the overall therapeutic efficacy.^49^ To explore the mechanisms underlying this contradictory role of CTLA-4 inhibition, we performed single-cell analysis, identifying seven CD4^+^ T cell subsets (Figures 6A and S10A; Table S10). We found that *CTLA-4*, the target gene of tremelimumab, is prominently expressed in Treg subsets, particularly in 4-1BB^+^ Tregs and proliferating Tregs (Figures 6B and S10B). Notably, the immunosuppressive 4-1BB^+^ Tregs^50^ exhibited highest clonal expansion, followed by the cytotoxic *CXCL13*^+^ CD4^+^ T cell subset (upregulating *IFNG*, *GZMB*, *TOX2*, and *KLRB1*) (Figure 6C), highlighting its key role in modulating immune responses during neoadjuvant ICI therapy. The proportion of 4-1BB^+^ Tregs was significantly increased with D + T treatment, but not with D treatment alone (Figure S10C), suggesting that this subset is a primary target of additional tremelimumab treatment. However, its increased proportion in non-responders but not in responders (Figure S10D) suggests a potential counteractive effect on early ICI responses.

The high clonal expansion of *CXCL13*^+^ CD4^+^ T cells likely accounts for their increased proportion following D + T treatment compared to D alone (Figure 6D), suggesting a role in enhancing anti-tumor effects. Cellular transition probability analysis confirmed a higher likelihood of naive CD4^+^ T cells differentiating into *CXCL13*^+^ CD4^+^ T cells, indicating favorable CD4^+^ T cell state changes in D + T (Figure 6E). However, overall cellular transition analysis revealed a rapid shift of CD4^+^ T cells toward immunosuppressive 4-1BB^+^ Tregs in D + T (Figure 6F). Notably, *CTLA-4* and *KLRB1* were among the top velocity genes (Table S11). Differential expression analysis between D + T and D revealed upregulation of interleukin-2 (IL-2)-signaling pathway genes, suggesting that IL-2 signaling drives the rapid transition of the CD4^+^ compartment toward immune suppression via 4-1BB^+^ Tregs (Figures 6G; Table S12). IL-2 signaling, known to promote Treg-mediated immune suppression,^51^ was significantly more upregulated in 4-1BB^+^ Tregs in D + T compared to D (Figure 6H). Glycolysis, known to exacerbate Treg suppressive functions, was not downregulated in 4-1BB^+^ Tregs under D + T, providing mechanistic insights into our observed clinical inefficiency^52^ (Figure 6I). In addition, CD8^+^ Tpex subsets exhibited a lower increase in stem-like signature (Figure S7L), reflecting an unfavorable immunosuppressive TME post-D + T. Overall, our findings suggest that while CTLA-4 blockade combined with PD-L1 inhibition expands *CXCL13*^+^ CD4^+^ T cells to enhance immunotherapy, IL-2-driven activation of suppressive 4-1BB^+^ Tregs counteracts this effect, ultimately limiting the benefit of dual immunotherapy. Thus, CTLA-4 blockade exerts a dual effect—enhancing anti-tumor immune responses while concurrently promoting immunosuppressive mechanisms.

### Neutrophil senescence and CCIs are involved in ICI responses

Myeloid and B cells within the tumor also influence ICI response. Through subclustering analysis, we identified eight myeloid cell subsets (Figure S11A; Table S13) and seven B cell subsets^53^ (Figures S12A and S12B; Table S14). Notably, the senescence module genes previously identified in malignant cells were prominently expressed in neutrophils (Figure S11B). Moreover, the senescence module score of neutrophils was significantly higher post-D+T compared to post-D (Figure S11C), suggesting a previously underappreciated role of neutrophils in immunotherapy. Furthermore, interferon-stimulated genes, including *IRF1*, *ISG20*, *IFIT2*, *IFIT3*, and *STAT3*, were among the DEGs in neutrophils. Gene set enrichment analysis using Reactome pathways^54^ revealed that neutrophil DEGs were significantly enriched in the interferon signaling pathway (*p* < 0.001, hypergeometric test). A therapy-induced neutrophil acquired an interferon gene signature, which is crucial for effective immunotherapy.^55^^,^^56^ Notably, combinatory immunotherapy enhances neutrophil senescence more than monotherapy, driven by interferon signaling. This highlights a mechanistic role of interferon-stimulated neutrophils in immunotherapy response through senescence induction.

Finally, we analyzed changes in CCIs across all immune cell subsets using MultiNicheNet (STAR Methods). Notably, interactions involving tumor-associated macrophages, Tstr, and 4-1BB^+^ Tregs were increased in non-responders but decreased in responders, suggesting their role in ICI resistance (Figure S11D). These findings underscore the importance of CCIs with immunosuppressive cell subsets in driving resistance to ICI therapy.

## Discussion

In this study, we identified key factors influencing the response to neoadjuvant immunotherapy with durvalumab (anti-PD-L1) monotherapy and its combination with tremelimumab (anti-CTLA-4) in HNSCC. We report enhanced activation of CD8^+^ T cell *TNFRSF9* and senescent malignancy in ICI responders, with the addition of possible neutrophil activation mediated by interferon pathways. In addition, we observed key immune suppressive activity in specific subset of *TNFRSF9*^+^ Tregs, which may be a suitable target in parallel with ICIs, instead of complete Treg depletion and inducing severe immune-related adverse events (irAEs). Lastly, our findings suggest that Tpex can be further divided into two subtypes, with one conversely contributing to ICI resistance. Strikingly the balance of these two subtypes of Tpex could predict therapy response across multiple tumor types.

Using NMF and a network-based computational approach, we identified core gene signatures in both malignant cells and CD8^+^ T cells. Our results highlight not only the pre-existing immunological states of CD8^+^ T cells that enhance ICI responsiveness but also the intrinsic properties of malignant cells that predispose them to ICI-induced clearance. Notably, while the senescence module was initially identified in malignant cells, we also observed its upregulation in neutrophils of the responder group. This aligns with prior reports of ICI-elicited neutrophil accumulation during successful immunotherapy.^55^^,^^56^ However, the association between combinatorial ICI treatment and neutrophil senescence requires further investigation.

Early post-ICI T cell expansion relative to baseline has been established as a strong indicator of response,^35^^,^^48^ as expanded samples exhibit heightened cellular cytotoxicity and enhanced immune cell interactions within the TME. However, in this neoadjuvant study, we found that early CD8^+^ T cell expansion did not correlate with tumor regression. In addition, tumor regression (and proportion of CXCL13^+^ Tex) was not correlated with the interval between ICI administration and surgery. This suggests that, in the neoadjuvant setting, successful immunotherapy may rely more on the pre-existing state of tumor-infiltrating T cells rather than “clonal replacement.” In addition, the expansion of bystander CD8^+^ T cells may obscure early T cell responses post-ICI treatment.

Our findings underscore the importance of identifying Tpex population in neoadjuvant immunotherapy, which was computationally feasible through single-cell TCR profiling. Notably, we observed that the ICI-resistant Tpex2 and ICI-responsive Tpex1 subpopulations could explain response heterogeneity across multiple cancer types, highlighting their pivotal roles in the pan-cancer ICI context. Based on these findings, we propose a predictor model that categorizes tumor-specific CD8^+^ T cells into Tpex1 (stem-like precursors associated with favorable ICI response), Tpex2, and Tex (cell states linked to ICI resistance). Evaluating the baseline ratio of these subsets may predict immune microenvironments conducive to successful ICI treatment.

### Limitations of the study

Our study has several limitations. The short observation window between baseline and post-ICI treatment may not capture the full spectrum of immune adaptation to checkpoint inhibitors. Some patients may require a longer duration for their immune environment to fully respond, restricting our findings to predisposed differences that define early ICI responses. Moreover, due to sample availability for translational analysis, we analyzed 29 patients with available single-cell transcriptomic data, although 45 patients were enrolled in the clinical study and there were no significant differences in baseline characteristics between patients with and without available samples for translational analysis (Table S15). Due to the known gender bias of head and neck cancer toward male, our cohort mostly consisted of male subjects. In addition, our results do not address long-term therapeutic effects, resistance mechanisms, or irAEs,^57^ which are critical for sustained ICI efficacy. Finally, future studies should integrate epigenetic and spatial transcriptional analyses of the TME to better characterize the differentiation trajectory of Tpex1 toward Tpex2. Understanding this suppressive transition will be essential for identifying mechanisms to sustain ICI responses and prevent tumor relapses.

## Resource availability

### Lead contact

Requests for further information and resources should be directed to and will be fulfilled by the lead contact, Hye Ryun Kim (nobelg@yuhs.ac).

### Materials availability

This study did not generate new, unique reagents.

### Data and code availability

• The raw and processed scRNA-seq and scTCR-seq data generated in this study have been deposited in the Gene Expression Omnibus database (https://www.nicbi.nlm.nih.gov/geo/) under GEO: GSE286827. Cell types identified in this study and their clonotypes (i.e., tumor-specific CD8^+^ T cells) are also made available.
• This paper does not report original code.
• Any additional information required to reanalyze the data reported in this paper is available from the lead contact upon request.

## Acknowledgments

This work was supported in part by the 10.13039/501100003725National Research Foundation funded by the Ministry of Science and ICT (2021R1A2C2094629, RS-2023-00261820, RS-2025-02214844, and RS-2022-CC125144 to H.R.K.; 2022M3A9F3016364 and 2022R1A2C1092062 to I.L.; and RS-2024-00348654 to C.G.K.); Brain Korea 21 (BK21) FOUR program; the Technology Innovation Program (20022947 to H.R.K.) funded by the MOTIE, Ministry of health and welfare (RS-2024-00411768 to C.G.K.); 10.13039/501100004082Korean Foundation for Cancer Research (9 CB-2022-C-1 to H.R.K.); and 10.13039/501100008005Yonsei University College of Medicine (4-2023-1187 to C.G.K.). This work was supported by the Yonsei Fellow Program, funded by Lee Youn Jae. Investigators who initiated the clinical trial were kindly supported by AstraZeneca. The funder had no role in the study design, data collection, analyses, and interpretation of data; in the writing of the report; or in the decision to submit the article for publication.

## Author contributions

H.R.K., I.L., Y.W.K., and M.H.H. conceived and designed the study. J.C. performed the bioinformatics analysis of single-cell omics data and formulated the study hypothesis under the supervision of I.L. C.G.K. contributed to patient enrollment in the clinical trial, clinical data collection, clinical data analysis, and correlation with genomic data. N.S.S. contributed to patient enrollment in the clinical trial, surgical procedures, clinical data collection, sample collection, and clinical data analysis. G.K., W.S., Jaehyung Kim, S.G., and Jeongah Kim performed sample preparation and experiments. D.K. contributed to patient enrollment in the clinical trial and sample collection. Jinna Kim contributed to radiological assessment and generation of clinical data from trial participants. S.O.Y. contributed to pathological assessment and generation of clinical data from trial participants. Y.J. and H.B.L. assisted with the bioinformatics analyses. E.S. and S.B. assisted in data collection. J.C., C.G.K., and N.S.S. wrote the original draft of the manuscript. All authors contributed to the editing of the manuscript.

## Declaration of interests

The authors declare no competing interests.

## STAR★Methods

### Key resources table

**Table:** 

REAGENT or RESOURCE	SOURCE	IDENTIFIER
**Antibodies**		

4-1BB/CD137/TNFRSF9 (D2Z4Y) Rabbit mAb	CST	Cat# 34594S
CD137 Ligand (4-1BB Ligand) Monoclonal Antibody (5G11)	Invitrogen	Cat# 14-9056-82
SignalStain IHC Dual staining Kit	CST	Cat# 36084

**Critical commercial assays**		

Genetle MACS dissociator	Miltenyi Biotec.	Cat# 130-093-235
Human Tumor Dissociation Kit	Miltenyi Biotec.	Cat# 130-095-929
Chromium Single cell 3′ Reagent Kit v3	10x Genomics	Cat# 1000075
Chromium Next GEM single cell 5p RNA library v1.2	10x Genomics	Cat# 10000265
Chromium Single Cell VDJ library v1	10x Genomics	Cat# 1000016
Illumina HiSeq X	Illumina	RRID: SCR_016385

**Deposited data**		

Raw and analyzed scRNA-seq and scTCRseq of tumor biopsies	This manuscript	GEO: GSE286827

**Software and algorithms**		

CellRanger	10X Genomics	Version 3
Seurat	https://satijalab.org/seurat/	Version 4
Harmony	https://github.com/immunogenomics/harmony	Version 1.2.1
Scanpy	https://github.com/scverse/scanpy	Version 1.7.2
enrichR	https://github.com/wjawaid/enrichR	Version 3.2
Slingshot	https://github.com/kstreet13/slingshot	Version 2.0.0
tradeSeq	https://github.com/statOmics/tradeSeq	Version 1.12.0
scVelo	https://github.com/theislab/scvelo/	Version 0.2.4
scDblFinder	https://github.com/plger/scDblFinder	Version 1.16.0
scATOMIC	https://github.com/abelson-lab/scATOMIC	Version 2.0.3
SCEVAN	https://github.com/AntonioDeFalco/SCEVAN	Version 1.0.0
cNMF	https://github.com/dylkot/cNMF	Version 1.7
ESTIMATE	https://github.com/KaiAragaki/tidyestimate	Version 1.1.1
GSVA	https://github.com/rcastelo/GSVA	Version 1.50.5
rpart	https://cran.r-project.org/package=rpart	Version 4.1.23
MultiNicheNet	https://github.com/saeyslab/multinichenetr	Version 2.0.0
scRepertoire	https://github.com/BorchLab/scRepertoire	Version 1.3.3
scHumanNet	https://github.com/netbiolab/scHumanNet	Version 1.0.0
Geneformer	https://huggingface.co/ctheodoris/Geneformer	Version 0.1.0
BioRender	https://www.biorender.com/	N/A

### Experimental model and study participant details

Pre- and/or post-treatment head and neck cancer tissues were obtained from 29 patients (27 male and 2 female) who underwent surgery between January 2019 and December 2020 at Yonsei University Severance Hospital. The cohort had a median age of 60 years 13 patients were enrolled in the monotherapy group, and 16 patients were enrolled in the combination therapy group. The studies were approved by the Institutional Review Board of Yonsei University Severance Hospital with IRB No 4–2018–0787. Written informed consent was obtained prior to enrollment and sample collection at Yonsei University Severance Hospital. The research conformed to the principles of the Helsinki Declaration.

### Method details

#### Generation of scRNA-seq and scTCR-seq data

Libraries were prepared as previously described method.^9^ Briefly, freshly harvested tumor tissues were processed using a gentleMACS dissociator (Miltenyi Biotec, Gladbach Bergisch, Germany, Cat#130-093-235) and the Human Tumor Dissociation Kit (Miltenyi Biotec, Cat#130-095-929) following the manufacturer’s protocol. Tissue-infiltrating lymphocytes were isolated using a Ficoll gradient (Sigma-Aldrich), and single-cell suspensions were counted using trypan blue.

Samples diluted to cell count of 10,000 with nuclease-free water were prepared using the Chromium controller, following the protocol outlined in the 10x Chromium Next GEM Single Cell 5′ v2 Cell Surface Protein User Guide (CG000330). With a master mix, diluted samples loaded alongside Single Cell 5′ Gel Beads and Partitioning Oil into a Next GEM Chip K. The resulting cDNA molecules were pooled and underwent PCR enrichment. The amplified cDNA was then size-selected to create 5′ Gene Expression libraries, V(D)J Enriched Libraries, and Cell Surface Protein libraries.

Quantification of the purified libraries was performed using qPCR following the KAPA qPCR Quantification Protocol Guide. Library quality was assessed with the Agilent 4200 TapeStation. Sequencing was conducted on the Illumina HiSeq platform according to the specified read length in the user guide.

#### Preprocessing and cell type annotation of scRNA-seq data

Quality control was performed for individual sequencing batches based on mitochondrial gene percentage, sequencing depth per cell, and the number of expressed feature genes per cell. Doublet cells were identified and removed using scDblFinder (v1.16.0),^58^ and cells with a read depth below 1,000 were excluded.

Malignant and immune cell compartment were delineated through a two-step process. First, Harmony(v1.2.1)^59^ was applied to correct for platform- and patient-specific batch effect across the dataset. Epithelial clusters expressing malignant cell marker genes (e.g., *KRT5*, *KRT14*, *KRT16*, *KRT17*)^60^ were identified using the standard normalization, dimension reduction, and Louvain clustering pipeline provided by the Seurat(v4.4.0) package.^61^ Next, to distinguish malignant from normal epithelial cells, we employed scATOMIC(v2.0.3),^20^ a pan-cancer reference-based classification tool that integrates CNV and expression profile using a pre-trained random forest model. Identified malignant cells underwent preprocessing with log normalization and dimension reduction. From the 4,000 variable genes extracted, those associated with ribosome, mitochondria, immunoglobulin, and T cell receptors were blaklisted.^62^ For downstream analysis, 35 principal components (PCs) were used for non-linear UMAP dimension reduction. Finally, SCEVAN(v1.0.0)^63^ was applied to malignant cells to assess chromosomal amplification and deletion.

For the remaining immune and stromal cells, SCT normalization^64^ was performed using 4,000 variable genes (excluding blacklisted genes) and 35 PCs. A resolution of 0.4 was chosen to define 20 broad immune and stromal cell subtypes. Marker genes were identified using *FindMarkers()* with the Wilcoxon rank-sum test and Benjamini-Hochberg P-value adjustment. For immune subclusters of CD8^+^ T cells, CD4^+^ T cells, Macrophages, and B cells, normalization was performed per sequencing batch using either log normalization via the *NormalizeData()* or SCT transformation via *SCTransform()*. Cell cycle gene effects were regressed out using *ScaleData()*. The top 4,000 variable genes were selected after applying the same blacklisting procedure across all immune subsets. Dimension reduction was performed using 30 PCs. To identify biologically relevant immune subclusters, we performed two rounds of clustering for each immune subset, initially matching the original broad selection criteria (CD8^+^ T, CD4^+^ T, macrophage, and B cells). Misclassified cells were removed, and the remaining cells were reprocessed. This step was particularly crucial for distinguishing CD4^+^ T cells from CD8^+^ T cells, as many ambiguous cells were mislabeled during the initial broad immune cell classification. The absolute count of all identified cell types analyzed in our study is given per sample in Table S1.

#### Identifying malignant cell programs

Malignant cell programs exhibit high heterogeneity depending on the origin of patients. To identify consistent cellular programs across patients, we partitioned malignant cell dataset into functional gene programs using consensus non-negative factorization (cNMF), following approaches inspired by Gavish et al.^10^ and Barkley et al*.*^11^

We leveraged the cNMF package^65^ to determine the optimal K parameter (ranging from 2 to 10) for each sequencing batch, using a diagnostic plot that evaluate data stability and error scores. For each malignant cell batch, cells with zero reads and genes with zero reads were filtered out. Sequencing batches with more than 100 remaining cells after filtering were included in the cNMF analysis. The *cnmf prepare* command was run with n-iter = 100 and numgenes = 4000. After examining the diagnostic plots generated for each batch, the K parameter was individually selected and used for the *cnmf consensus* command, with a density threshold of 0.01. Only coding genes from the Consensus Coding Sequences (CCDS) database^66^ were considered for the analysis.

The cNMF *Z* score output of variable genes ("over dispersed genes" identified by cNMF) was used to extract modules from individual samples. A gene was included in a sample-specific module if (1) its rank among cells was above the average and (2) it had the highest rank among the K modules. Using this approach, we identified 209 modules from 57 samples. To retain only modules with notable overlaps across samples, we filtered out patient-specific gene programs by calculating pairwise Jaccard indices. Modules were retained if they had a Jaccard index score of at least 0.05 with at least 50 other modules (approximately the top 25% of the data). This resulted in 70 modules that were consistently detected across multiple samples, which were used to construct the meta-module cancer program. A heatmap of the 70 modules was generated based on hypergeometric test P-values (-log_10_), using the entire CCDS coding gene space as the total gene space. The result confirmed significant overlap between modules across patients.

To define distinct functional meta-programs incorporating the filtered consensus modules, we developed a computational framework to construct non-overlapping gene sets from the 70 modules. First, the top 100 genes by *Z* score were extracted from each module to ensure equal weights across modules with varying gene sets. Next, a co-occurrence matrix was generated for each gene, representing the ratio of co-occurrence frequency to the sum of individual frequencies. The resulting adjacency matrix was then used to construct a gene-gene network. Non-overlapping network community detection was performed using the Louvain algorithm, with a resolution parameter of 1.2, resulting in nine meta-programs. The functional roles of each gene set were analyzed using gene set analysis (GSA) on KEGG, GOBP, and MsigDB databases. The genes within each of the nine meta-programs, sorted by degree centrality, are listed in Table S2. Meta-program names were assigned based on enriched functional terms from these databases and gene overlap with meta-program identified by Gavish et al.

#### Identification of meta-programs associated with ICI response

Meta-programs associated with ICI tumor regression were evaluated with the assumption that each malignant cell is driven by a single representative malignancy program. Meta-program scores were calculated for individual cells using the top 50 genes (ranked by degree centrality) with the *AddModuleScore()* function. Each cell was then assigned to one of the nine meta-programs based on its highest enrichment score. Pie charts were generated for individual patients and treatment response groups based on this classification. For each patient, the proportion of cells assigned to each meta-program was calculated. Pearson correlation coefficients were used to identify the meta-programs most strongly associated with ICI response. The "CC Translation" program showed the strongest negative correlation, indicating an association with ICI nonresponse, while "Epithelial Senescence" program showed the strongest positive correlation, indicating an association with ICI response.

To derive the senescence program signature genes, we manually reviewed the top central genes of the Epithelial senescence program for functions related to tumor suppression and/or immune system upregulation, based on prior research. We found that the transcription factor ELF3 (ranked 3rd by centrality) was strongly associated with age,^67^ as expected. PDZK1IP1 (ranked 5th by centrality) is supported in the literature as a tumor suppressor gene^68^^,^^69^ and was also part of the MP19 Epithelial Senescence program proposed by Gavish et al.^10^ Upon examining the direct network neighbors of PDZK1IP1, we identified genes such as *TNFSF10*, *OAS1*, *CXCL17* and others that are known to be involved in immune system regulation. Based on these findings, we defined this subset of genes as the "senescence program" and used this term throughout the manuscript. The enrichment of the senescence program in individual cells was calculated using the *AddModuleScore()* function. The variance of the enrichment scores was calculated with the *var()* function from base R, and the difference of variance (pre-vs. post-ICI treatment) was statistically tested using the *leveneTest()* function from *car* package.

#### Assessment of senescence program for ICI response predictions

To assess the effect of signature genes in bulk RNA cohorts, we collected publicly available studies from the Tumor Immunotherapy Gene Expression Resource (TIGER).^70^ Immune signatures known to be associated with ICI response were obtained from individual studies.^71^ In addition to those in the TIGER database, we gathered data from three other studies: Hsu 2021 (HCC),^72^ Ende 2021 (EAC),^73^ Rose 2021 (UC).^74^ All cohorts were further divided into their ICI treatment group (e.g., anti-CTLA-4, anti-PD-1). After data collection, we applied the following filtering criteria: datasets with fewer than 10,000 genes were excluded; samples with an unknown response status (e.g., UNK) were discarded, and only those labeled as "responder" or "non-responder" were retained. We selected 16,247 coding genes as the total gene space, assigning a value of 0 for genes not present in a sample. Datasets with fewer than 30 samples were also excluded. In addition, we removed VanAllen and Braun data, as they showed no predictive power for any of the signatures tested (average AUROC approximately 0.5). As a result, we proceeded with 8 bulk cohorts, comprising a total of 549 samples.

Eight random forest (RF) classifiers with a maximum tree depth of 5 were constructed with 7-fold cross validation, where the classifier was trained on 7 studies and tested on 1 other study. We selected Ayer et al. gene set^21^ as the representative immune gene set, as it has consistently demonstrated strong predictive performance across multiple independent bulk RNA-seq studies. The ESTIMATE algorithm^24^ was used to infer stromal, immune, and tumor purity. Stromal score, tumor purity, and Ayer module score were used as the predictor for each of the 8 RF models, which were compared to models that included the senescence program as an additional predictor. The senescence program score and Ayers signature were measured using GSVA^75^ and the tree models were constructed with the rpart package in R (v4.1.23). The final ensemble model was created by averaging the probability scores from the eight individual RF classifiers, which was used to assess the performance changes when adding the senescence program as a predictor. We observed that 7 out of 8 individual RF models showed similar or improved performance when the senescence program was included as a predictor.

#### Composition analysis of immune cells with Pearson residual

To investigate proportional changes between sample groups (e.g., Mono vs. Combination therapy) across cell subsets defined by clustering, we evaluated deviations in the observed cell count for each group from the expected count for a subset using Pearson residual ($r_{ij})$:

$$r_{ij}=\frac{O_{ij}-E_{ij}}{\sqrt{E_{ij}}}\text{,}$$

where *i* and *j* represent indices for each group and cell subsets, respectively, and *O* and *E* represent the observed and expected cell counts, respectively. The expected cell count for a group *i* of a subset *j* was calculated by the following equation:

$$E_{ij}=\frac{T_{i}}{T_{tot}}\times T_{j}\text{,}$$

where *T*_tot_, *T*_*i*_*,* and *T*_*j*_ represent total cell count for the entire dataset, total cell count for a group *i*, and total cell count for a subset *j*, respectively. The advantage of the Pearson residual is that the sign of the residual indicates the direction of the difference of the observed count from the expected count (i.e., positive for augmentation and negative for depletion compared to expected count). Pearson residual (*r*) follows an approximately normal distribution; thus, scores larger than 2 or smaller than −2 are significant by *p* < 0.05. We considered only cell subsets with *r* > 3.5 (augmentation) and *r* < −3.5 (depletion) for follow-up functional interpretation. We visualized the results of the goodness of fit test using a mosaic plot, in which subsets with deviation in observed cell counts from the expected cell counts are indicated by blue (augmentation), red (depletion), or gray (no significant change) colors. The goodness of fit for all subsets was also evaluated by the chi-square statistic (*p* value).

#### Identification of marker genes for each cell type

To minimize technical artifacts associated with sequencing depth across patients and platforms, we applied SCT normalization to identify marker genes for each cell type. Marker gene identification was performed using the *FindAllMarkers()* function from the Seurat package with default parameters. For differential expression analysis comparing ICI response and treatment arms, we used the *FindMarkers()* function with default parameters.

Given the large sample size (*n* = 57) and the multi-subject nature of our study, we applied a negative binomial mixed model (NEBULA)^34^ proposed by He et al. to identify genes associated with ICI response and treatment arms. NEBULA accounts for both cell-level and subject-level overdispersion, reducing false-positive findings compared to the naive Wilcoxon rank sum test commonly used in single-cell studies at the patient level.^76^ We specifically used NEBULA-HL, which is based on the standard h-likelihood method as it demonstrated the highest performance in an independent benchmark study.^77^ Assuming that each predictor independently effects gene expression, we modeled gene expression using the following formula:

Expression∼celltype+ICItime+ICItype+ICIresponse,

where individual gene expression is modeled based on ICI time indicating pre or post treatment, cell type indicating the subclusters of celltypes (e.g., Tstr of CD8^+^ T cells), ICI type indicating mono (D) or combination (D + T) therapy, and ICI response indicating the responder (R) or non-responder (NR) label. A summary of significant genes (*p* < 0.05) identified by NEBULA-HL is provided in Table S5.

#### Differentially expressed genes along cell lineages

To identify DEGs between responder and non-responder cell groups within the activated CD8^+^ T cell lineages (from Tpex to HSP and Tpex to Tex), we used the *ConditionTest()* function from tradeSeq (v1.12.0).^78^ For log2 fold change (*l2fc*), a threshold of 1 was set to detect genes with at least a 2-fold change. The Wald test was used to assess significance, and P-values were adjusted using the Benjamini-Hochberg method, retaining genes with *q* ≤ 0.05. The significance determined by the Wald test provided evidence of gene expression differences associated with ICI response along the identified tumor-reactive CD8^+^ T cell trajectory.

#### RNA velocity analysis

RNA velocity was analyzed using the scVelo^14^ package (v0.2.4). Briefly, scVelo quantifies the time-dependent relationship between unspliced and spliced mRNA without assuming a steady state. RNA velocity was inferred using the "dynamic" model, with n_pcs = 30 and n_neighbors = 30 specified in the *scv.pp.moments()* function. The transition probability matrix was computed using *scv.utils.get_transition_matrix()* function and subsequently normalized. For each "start" cell, the transition probabilities of all target cells were summed to account for differences in cell abundance. Instances of zero probabilities were excluded. The resulting probability matrix was visualized as a density plot for selected T cell subsets using the ggplot2 package in R. Significant differences between density distributions were assessed using a two-sided Kolmogorov-Smirnov test.

#### Stemness signature analysis

The stemness signature genes from Sade-Feldman et al.^47^ were used to score tumor-reactive T cells using the *AddModuleScore()* function in the Seurat package. As proposed by Tirosh et al.*,*^79^ the average expression of the stemness signature genes was computed and adjusted by subtracting the aggregated expression of control feature sets. Control feature sets were defined by binning all analyzed genes into 25 bins based on the aggregate expression levels. For each gene in the stemness signature set, 100 genes were selected from the same expression bin to construct a control gene set. This approach ensures that the control feature sets have a comparable expression distribution to the stemness gene set (or any gene set under investigation). In addition, since each gene in the stemness signature set is matched with 100 control genes, the control gene set is 100-fold larger, making its average expression comparable to the mean of 100 randomly selected gene sets of the same size as the stemness signature set.

#### Pseudotime analysis

Pseudotime analysis was performed using Slingshot^80^ (v2.0.0) and tradeSeq^78^ (v1.12.0). Log normalized gene expression values were used to infer pseudotime, with dimensionality reduction performed using Harmony, followed by UMAP embedding. For CD8^+^ T cells, the *start.clus* parameter was set to Tpex1, and *end.cluster* parameter was set to Tex. To identify genes associated with pseudotime, the *evaluateK()* function from the tradeSeq package was used to determine the optimal *k* parameter. Based on this evaluation, nknots = 6 was selected for *fitGAM()*.

#### Cell-cell interaction analysis

Similar to the DEG analysis, the multiple-sample and multi-condition design of our study posed challenges in calculating differential cell-cell interactions. To address this, we utilized the MultiNicheNet package (v.2.0.0) (https://github.com/saeyslab/multinichenetr). MultiNicheNet accounts for inter-sample heterogeneity using mixed models and pseudobulk aggregation.^81^ Cell-cell interactions were considered differential between groups if they met the following parameter threshold: min_cells = 10, min_sample_prop = 0.5, fraction_cutoff = 0.05, logFC_threshold = 0.5, scenario = "regular". For cases where the distribution of DE *p*-values was not uniform, we set empirical_pval = TRUE, following the recommendations of the MultiNicheNet developers. Among CD8^+^ T cell subclusters, the TNFSF9-TNFRSF9 interaction between *CXCL13*^*+*^ Tex cells and other CD8^+^ subsets ranked within top 10 interactions, as determined using the *get_top_n_lr_pairs()* function, with rank_per_group = TRUE for post responder group. We only considered upregulated interactions in the post-ICI treatment group, as "downregulation" of cell-cell interactions can be misleading due to variation in baseline expression across human samples. Notably, an observed decrease in interaction post-treatment may also be interpreted as an increase in pre-treatment depending on the reference baseline.

#### Dual immunohistochemistry staining

Paraffin-embedded post-surgery tumors were sectioned by 2–4 μm thick slice on coated-slide, then de-paraffinized, and rehydrated by gradient ethanol solutions. Heat-induced antigen retrieval was performed with low pH citrate buffer. Samples were then incubated with anti-human CD137 (gene symbol; TNFRSF9, CST, Cat#34594S, Clone#D2Z4Y; diluted 1:50), or anti-human 4-1BB ligand (gene symbol; TNFSF9, Invitrogen, Cat#14-9056-82, Clone#5G11; diluted 1:150). Secondary antibody incubation and chromogenic reaction were performed with SignalStain IHC Dual Staining Kit (AP, Rabbit, Red/HRP, Mouse, Brown, CST, Cat#36084) according to the manufacturer’s protocol. The images were acquired by slide scanning microscope (Olympus, BX43).

#### Analysis of tumor-specific CD8^+^ T cell subsets

To analyze pan-cancer tumor-specific CD8^+^ T cell subsets and their relationship with immunotherapy response, we performed scRNA-seq analysis using datasets from multiple cohorts, focusing exclusively on tumor biopsy samples. The analyzed datasets included GSE123813,^82^ SRP308561,^83^ GSE120575,^47^ GSE176021,^84^ GSE179994,^15^ GSE207422,^85^ SCP1288,^86^ and GSE169246,^87^ covering cancer types such as basal cell carcinoma (BCC), non-small cell lung cancer (NSCLC), clear cell renal cell carcinoma (ccRCC), melanoma (Mela), squamous cell carcinoma (SCC), and triple-negative breast cancer (TNBC).

For some datasets (GSE123813, SRP308561, GSE120575, GSE207422, and GSE169246), pre-processed and quality-controlled data from the respective studies were used. However, additional quality control (QC) steps were required for the remaining datasets. Using the ddqc R package,^88^ QC metrics were computed with the *ddqc.metrics()* function, and the filtering thresholds were adjusted from the default value of 2 to 1 to enforce stricter cell selection criteria. Doublets were identified using scDblFinder,^58^ with a uniform doublet rate of 0.06.

Gene symbols were standardized to GRCh38-based official symbols using the limma and org.Hs.e.g.,.db R packages to ensure consistency across datasets. The *alias2SymbolTable()* function was used to map gene aliases to their official symbols, enhancing annotation accuracy.

CD8^+^ T cells were initially identified using Celltypist,^89^ with the prediction label "T cells" extracted from the collected datasets. To obtain tumor-specific CD8^+^ TILs, we applied the following filtering criteria: cells with *CD8A* expression > 0, *CD4* expression = 0, and *ITGAE* and *ENTPD1* expression > 0.^32^ For label transferring, the Seurat integration method was used with the *TransferData()* function and default parameters. For each cell, a score for the input query cell type was calculated, and the maximum score was selected as the final predicted cell type.

#### scTCR-seq data analysis and identification of tumor-reactive T cells

For the analysis of TCR sequence reads, we used 10x output filtered_contig_annotation.csv files. Barcodes were mapped to the GEX transcriptomes and included as columns in the metadata slots of Seurat objects. Clonotype was defined using the "CTstrict" method from the scRepertoire (v1.3.3) package (https://github.com/BorchLab/scRepertoire), which combines the V gene with a >85% normalized Levenshtein distance of the CDR3 region sequence. The expansion status of clonetypes was determined using the *table()* function in base R (v4.0.3). Expanded clonotypes were classified as those containing either five or more cells belonging to the same clonotype, or fewer than five but more than one cell with the same clonetype.

Tumor-reactive T cell extraction, leveraging TCR information, was inspired by Liu et al*.*^15^ Among CD8^+^ T cells, we separately identified cells with identical clones from the exhausted subset (*CXCL13*^*+*^ Tex cells), under the assumption that these represent cells that confidently encountered tumor antigens. Including *CXCL13*^*+*^ Tex cells, we extracted these T cells and preprocessed the data with Seurat pipeline, allowing us to categorize tumor-reactive T cells into various cellular states.

#### Network analysis of *CXCL13*^*+*^ Tex cells

False positives from standard DEG analysis, particularly when working with large patient cohorts in scRNA-seq data, can complicate the interpretation of results. To extract biologically relevant genes associated with ICI response, we leveraged scHumanNet,^13^ a cell-type specific gene network modeling method from our previous work. scHumanNet complements DEG analysis by identifying genes with statistically distinct network topology between different groups within a given cell type. Genes exhibiting differential hubness often reflect critical functional differences, and we hypothesized that differential hub genes in *CXCL13*^*+*^ Tex cells between responders and non-responders would reveal key functional differences related to varied ICI responses, particularly in neoadjuvant settings. This hypothesis is supported by the observation of persistent clonal expansion (clones present in both pre- and post-ICI conditions), which account for most of the expanded T cells in our dataset and has also been observed by others.^41^ These findings suggest that pre-existing resident CD8^+^ T cells are the key cell type involved in neoadjuvant ICI response.

We focused on genes modeled in both the response and non-response network (pre-ICI treatment), as group specific genes did not show significant enrichment of T cell functions. We hypothesized that genes with decreased network centrality in non-responders compared to responders are critically dysregulated and associated with the maintenance of ICI response. Differences in centrality ranks have previously been shown to identify biologically important genes.^90^ In the responder *CXCL13*^*+*^ CD8 T cells, we identified a subcommunity containing 129 genes highly relevant to T cell functions. Within this subcommunity, we extracted 43 genes with higher centrality (percentile rank to normalize for node size difference) compared to non-responder network. By ranking these genes based on their network centrality, we defined the top 30 genes as the "responder signature", which is summarized in Table S6.

*CXCL13*^*+*^ Tex cell type specific networks were constructed for each patient. To assess the similarity of networks, we first constructed a binary vector by taking the union of all nodes from each patient-specific network as the total vector space. We then calculated the Euclidean distance between nodes, assigning a value of 1 in the node existed in the network and 0 if it did not. The edges of the network are determined by the modeled nodes, based on the reference interactome algorithm implemented in scHumanNet.

#### Geneformer foundation model fine-tuning analysis

We leveraged the Geneformer foundation model^12^ to assess the effect of signature genes we derived in the context of neoadjuvant immunotherapy response. The model was pretrained with data from 30 million cells using a 12-layer transformer architecture. We fine-tuned this model with our input data, which comprised of 172,478 immune cells (macrophages, B cells and T cells). Before tokenizing the data, we filtered the genes to include only coding genes from the CCDS database (version 20221007). Fine-tuning was performed by retraining the last 4 layers of the Geneformer model, with the objective of classifying cells as responders versus non-responders. Changing the last n-layer parameter (freeze_layer) had minimal effect on the overall classification performance, with the total AUC achieving approximately 0.99. Hyperparameters included a max input size of 2,048, max learning rate of 0.00005, warmup steps of 10,000. 10 epoch, a linear scheduler, and 0.001 weight decay. We used the AdamW stochastic optimizer. For each gene, we modeled the embedding shift of *CXCL13*^*+*^ Tex cells from the responder state to the non-responder state by removing the expression rank. We used 3,804 housekeeping genes, downloaded from Eisenberg et al.*,*^91^ as control. Additionally, we randomly sampled 100 genes from the resulting perturbation output (*perturb_data()*) of the Geneformer package for comparison. A positive value of the cosine similarity shift indicates a shift of immune cells toward the non-responder state.

### Quantification and statistical analysis

Statistical analyses were performed using R v4.0.3. The statistical methods and details of data presentation are provided in the figure legends. Software packages and their versions used for each analysis are described in the Methods section. Statistical significance was defined as *p*-values or FDR <0.05 (∗, *p* < 0.05; ∗∗, *p* < 0.01; ∗∗∗, *p* < 0.001; and ∗∗∗∗, *p* < 0.0001). The number of samples or independent experiments is indicated in the main text or figure legends.

#### Additional resources

This trial was registered at ClinicalTrials.gov: NCT03737968.

## Supplemental information

Document S1. Figures S1–S11

Table S1. Clinical metadata of samples and their analyzed cell types (counts)

Table S2. Genes in the identified 9 meta-programs sorted by degree centrality

Table S3. Senescence module signature genes

Table S4. Top 50 DEGs expressed above 0.25 log2 fold genes in CD8^+^ T subclusters

Table S5. DEGs identified by NEBULA-HL for each cell types associated with ICI response and time

Table S6. Responder network signature genes

Table S7. Geneformer *in silico* gene perturbation in the pre-ICI CXCL13+ Tex cells

Table S8. Tumor-specific CD8^+^ T subcluster markers

Table S9. Significantly associated genes along lineage 1 of tumor-specific CD8^+^ T cells (Tpex to Tex) by tradeSeq

Table S10. Top 50 DEGs expressed above 0.25 log2 fold genes in CD4^+^ T subclusters

Table S11. CD4^+^ T subcluster top 100 velocity genes (D & D + T)

Table S12. DEG statistics of 4-1BB + Treg DEGs D + T vs. D

Table S13. Top 50 DEGs expressed above 0.25 log2 fold genes in tumor-associated macrophage subclusters

Table S14. Top 50 DEGs expressed above 0.25 log2 fold genes in B cell subclusters

Table S15. Baseline and treatment characteristics according to the availability of single-cell transcriptomic data

Document S2. Article plus supplemental information
