# Supplementary material for: 4-1BB+ Tregs and inhibitory progenitor exhausted T cells confer resistance to anti-PD-L1 and anti-CTLA-4 combination therapy
Source: Cell Rep Med. 2025 Oct 3;6(10):102408. doi: 10.1016/j.xcrm.2025.102408 (PMC12629806; doi:10.1016/j.xcrm.2025.102408)
Supplement: Document S1. Figures S1–S11 [file mmc1.pdf]

Cell Reports Medicine, Volume *6*

Supplemental information

4-1BB$^+$ Tregs and inhibitory progenitor exhausted T cells confer resistance to anti-PD-L1 and anti-CTLA-4 combination therapy

Junha Cha, Chang Gon Kim, Nam Suk Sim, Gamin Kim, Wonrak Son, Dahee Kim, Yurim Jung, Hyun Jun Hong, Hae Been Lee, Jaehyung Kim, Jinna Kim, Sun Och Yoon, Seokhyeong Go, Jeongah Kim, Euijung Seong, Seungbyn Baek, Kyung Hwan Kim, Min Hee Hong, Yoon Woo Koh, Insuk Lee, and Hye Ryun Kim

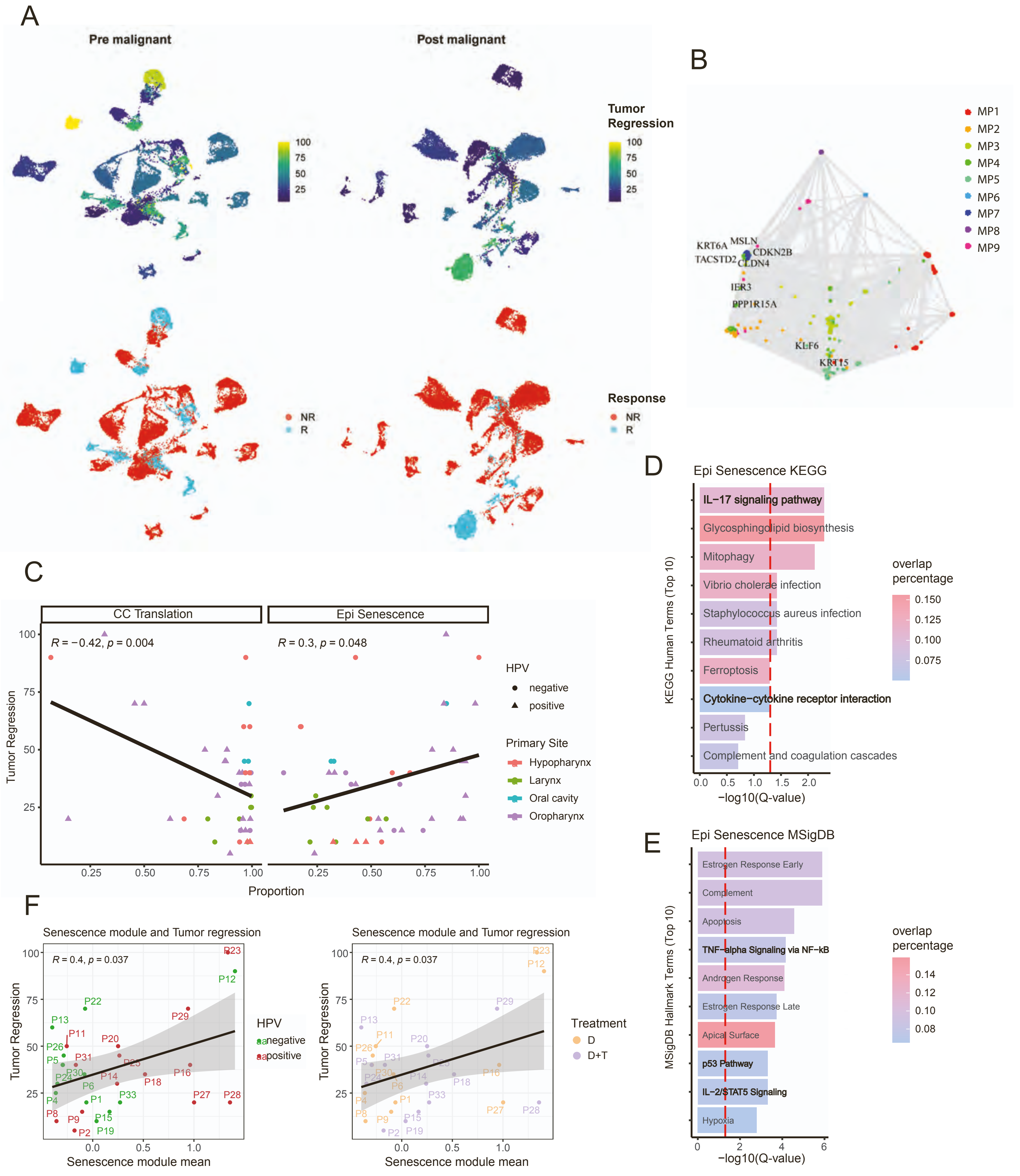

Figure S1 | Meta-program identified in malignant cells associated with tumor regression. Related to Figure 2. A. UMAP of pre-ICI (left) and post-ICI (right) malignant cells, colored by their tumor regression values (top) and their response status (bottom, tumor regression 50 percent or over). **B.** Co-occurrence network of genes constructed from patient specific non-negative factorization (NMF) modules. Colors indicate nine non-overlapping sub-communities (meta program) determined by Louvain clustering. **C.** Scatter plot of all malignant cells by samples, labeled by their primary site and HPV status. Proportion of Cell Cycle Translation and Epithelial Senescence meta program and their tumor regression values. Pearson correlation coefficient and its P-value is depicted. **D-E.** Gene set enrichment analysis of Epithelial Senescence meta program showing top 10 enriched term in KEGG **(D)** and MsigDB **(E)**. The red dashed line indicates adjusted P-value (Benjamini-Hochberg) of 0.05. **F.** Scatter plot of average senescence module score for each sample at baseline, and their tumor regression value colored by HPV status (left) and treatment group (right). Pearson correlation coefficient and its P-value are depicted.

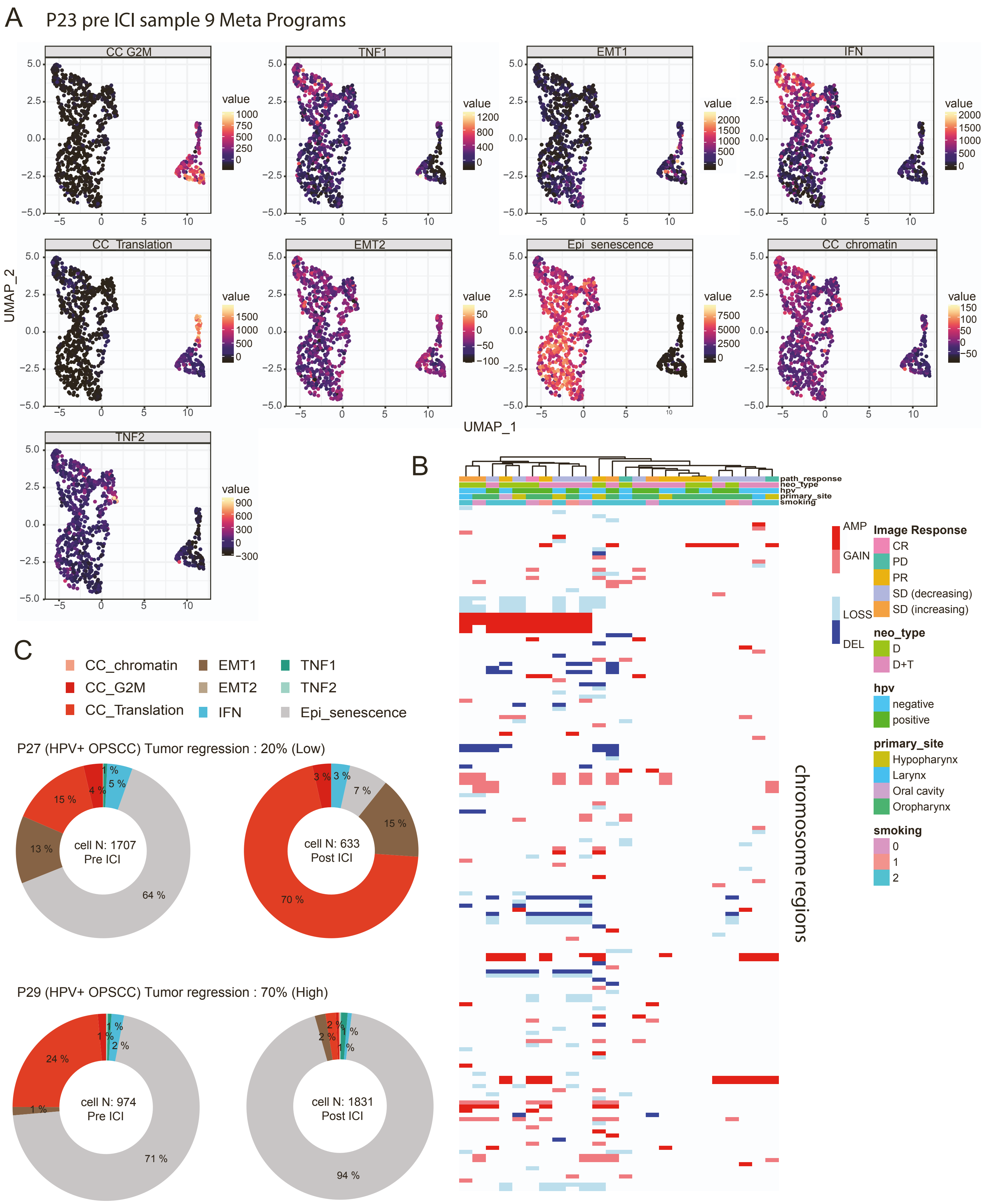

Figure S2 | Meta program expression and enrichment in tumors. Related to Figure 2. A. Example of distinct meta program scores in UMAP dimensions (mean TPM value output by cNMF) for sample P23, pre-ICI malignant cells. **B.** Gene amplification (gain) and deletion (loss) inferred by SCEVAN for each malignant sample. Inferred copy number variation is shown for specific chromosome regions (y axis). Samples are labeled for their pathological response, treatment group (neo_type, 1: monotherapy, 2: combination therapy), human papilloma virus (hpv) status, primary site, and smoking (0: none, 1: former smoker, 2: current smoker). **C.** Example pie chart for proportion of identified 9 meta-programs, in pre- and post-ICI malignant cells for HPV-positive OPSCC sample with low tumor regression (top) and high tumor regression (bottom).

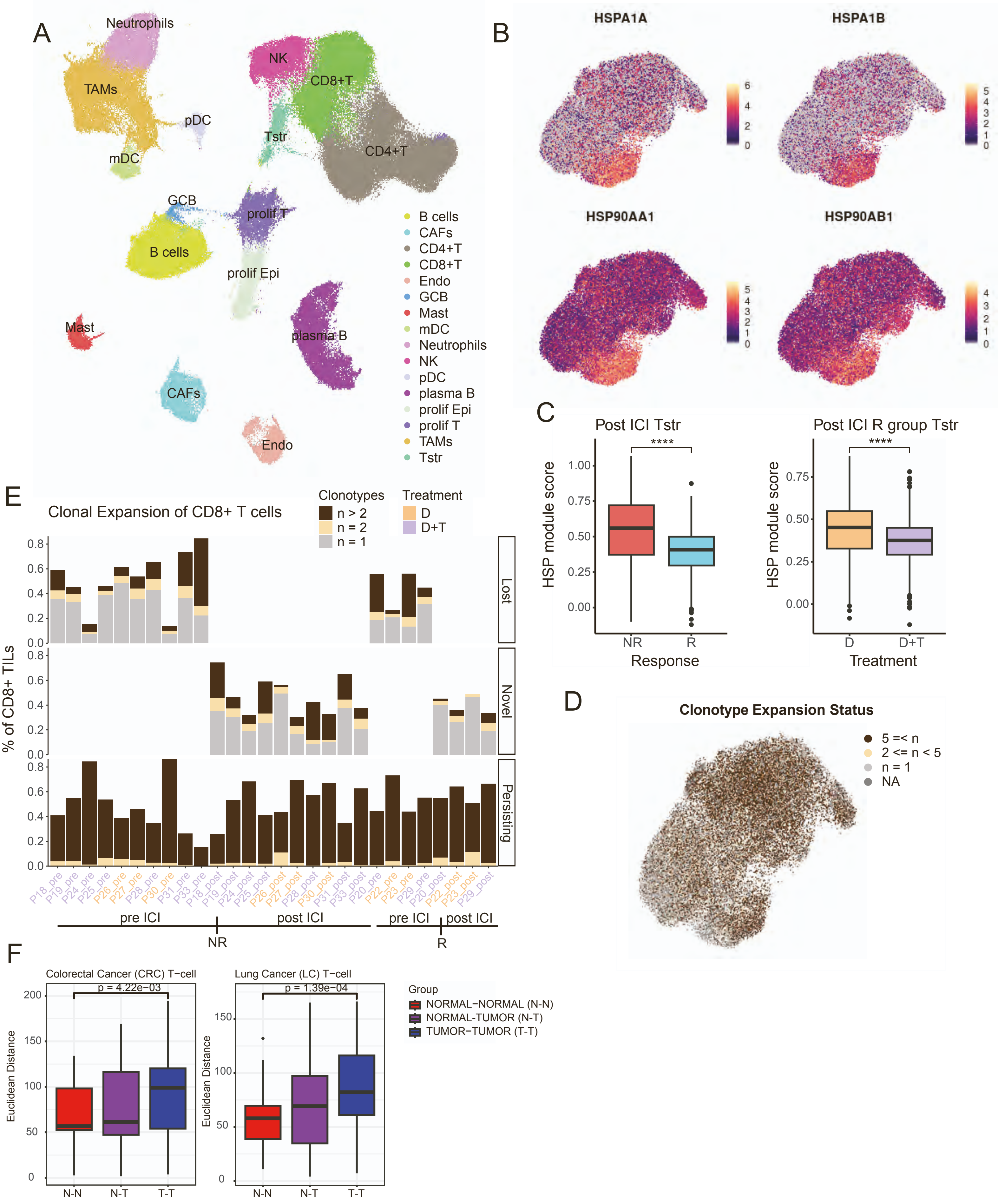

Figure S3 | CD8+ T subcluster analysis of stress response, clonotypes, and network structure. Related to Figure 3. A. UMAP of all major immune cells identified. **B.** Feature plot of four heat shock protein genes in CD8+ T cell UMAP dimensions. **C.** Boxplot of HSP module score derived from significantly upregulated genes specific to Tstr, comparing response group (left) and treatment group (right). P-values are calculated via Wilcoxon rank sum test. **D.** UMAP of CD8+ T cells and their clonotype expansion status label. NA labeled cells do not have TCR information. **E.** Bar plot of clonotype percentage colored by their expansion status. Clones are divided into three categories; lost after ICI (top, "Lost") gained after ICI (middle, "Novel") and existing in both conditions (bottom, "Persisting"). Samples in the x-axis are ordered by their response and treatment time and colored by their treatment group. **F.** Similarity of patient-specific T cell network nodes calculated with pairwise Euclidean distance from adjacency matrix of the union gene set. P-values were calculated via two-sided t-test. Distances are measured within group (tumor-tumor, normal-normal) and between group (tumor-normal). In the Qiao et al. dataset, lung cancer and colorectal cancer cohort were chosen as their cell number per patient was adequate.

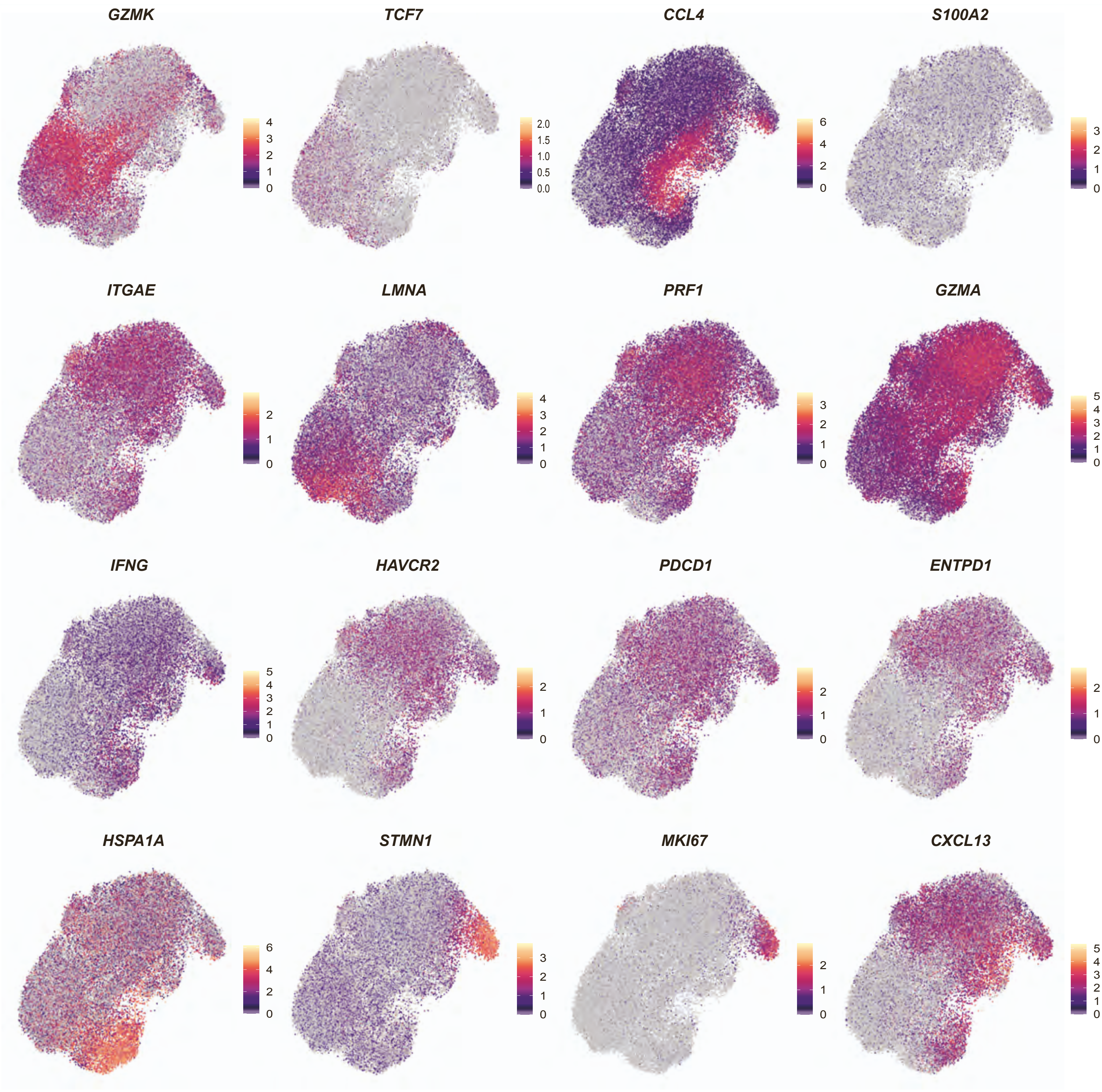

Figure S4 | Key gene expressions in CD8+ T cells. Related to Figure 3. Feature plot of representative genes specifically upregulated in CD8+ T cell subclusters.

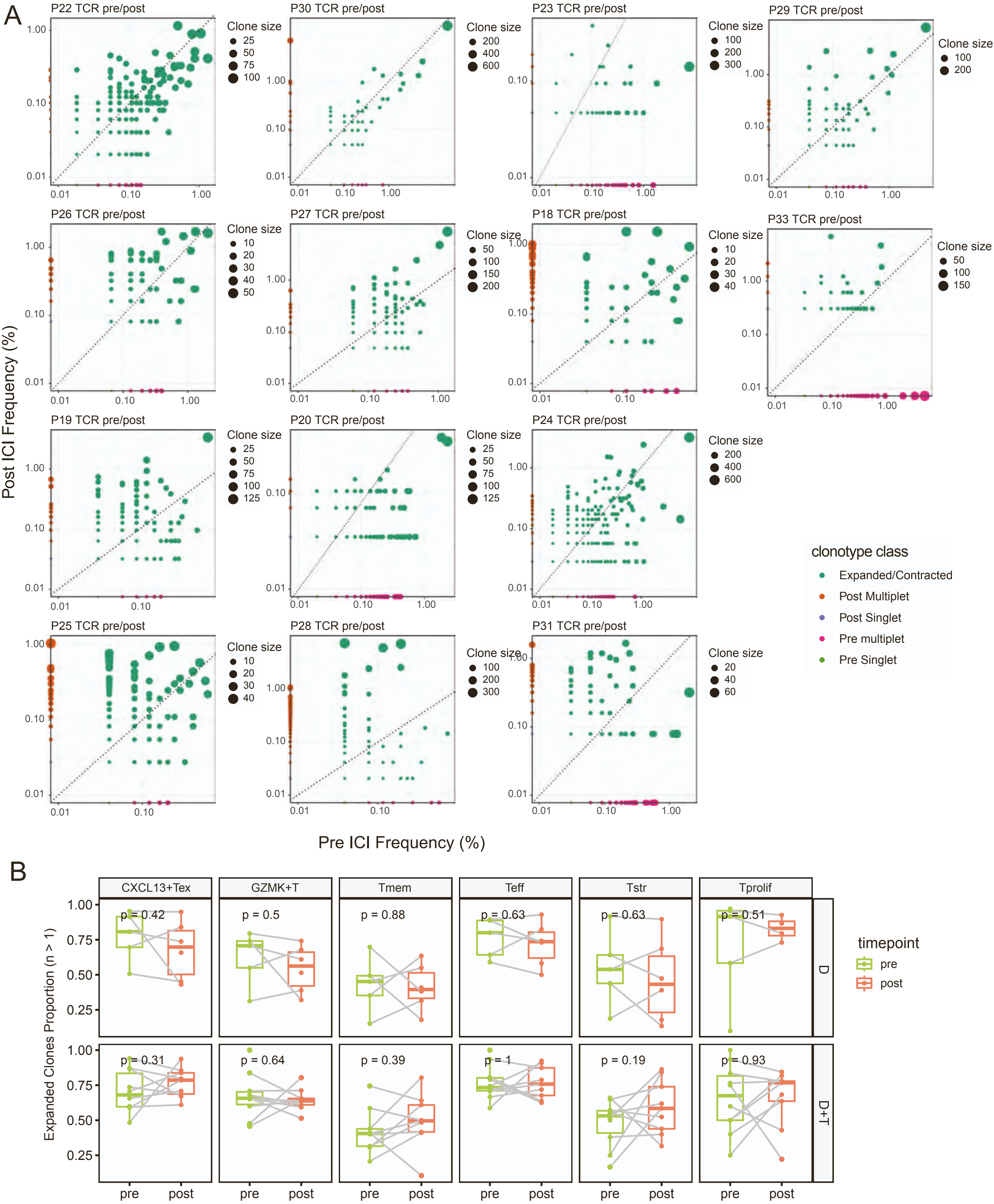

Figure S5 | Clonotype expansion in CD8+ T cells. Related to Figure 3. **A.** Scatter plots comparing clone frequencies pre- and post-ICI treatment measured by scTCR-seq. Colors indicate clone class and the dashed line represent the y = x axis dividing expanded or contracted clones post ICI treatment. Dot sizes indicate the absolute number of the TCR clones. **B.** Clone proportion for paired samples, in each CD8+ T cell sub clusters divided by their treatment group (D or D + T). P-value calculated via two-sided t-test.

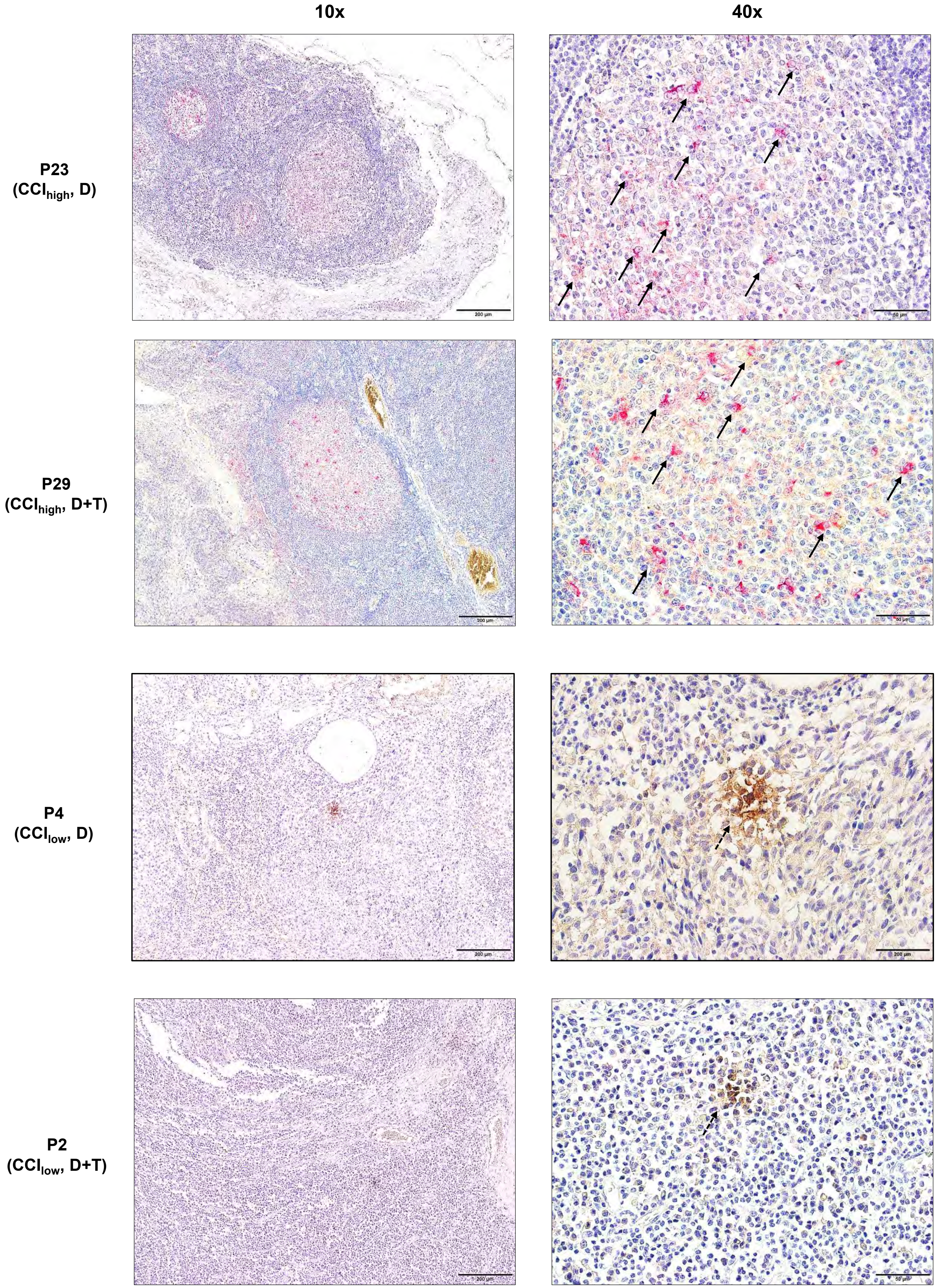

Figure S6 | Immunohistochemistry results within CD8+ T cells. Related to Figure 3. CD137 (TNFRSF9) expression co-localization with 4-1BBL (TNFSF9) in representative immunohistochemistry images (10x, 40x) showing CD137 (TNFRSF9, red), and 4-1BBL (TNFSF9, brown) in post tumor. Line arrow indicates TNFRSF9-TNFSF9 interaction, dashed arrow indicated TNFRSF9, or TNFSF9 only. Patients are indicated with cell-cell interaction level, and neoadjuvant ICI treatment. CCI; cell-cell interaction, D; durvalumab, D+T; durvalumab + tremelimumab.

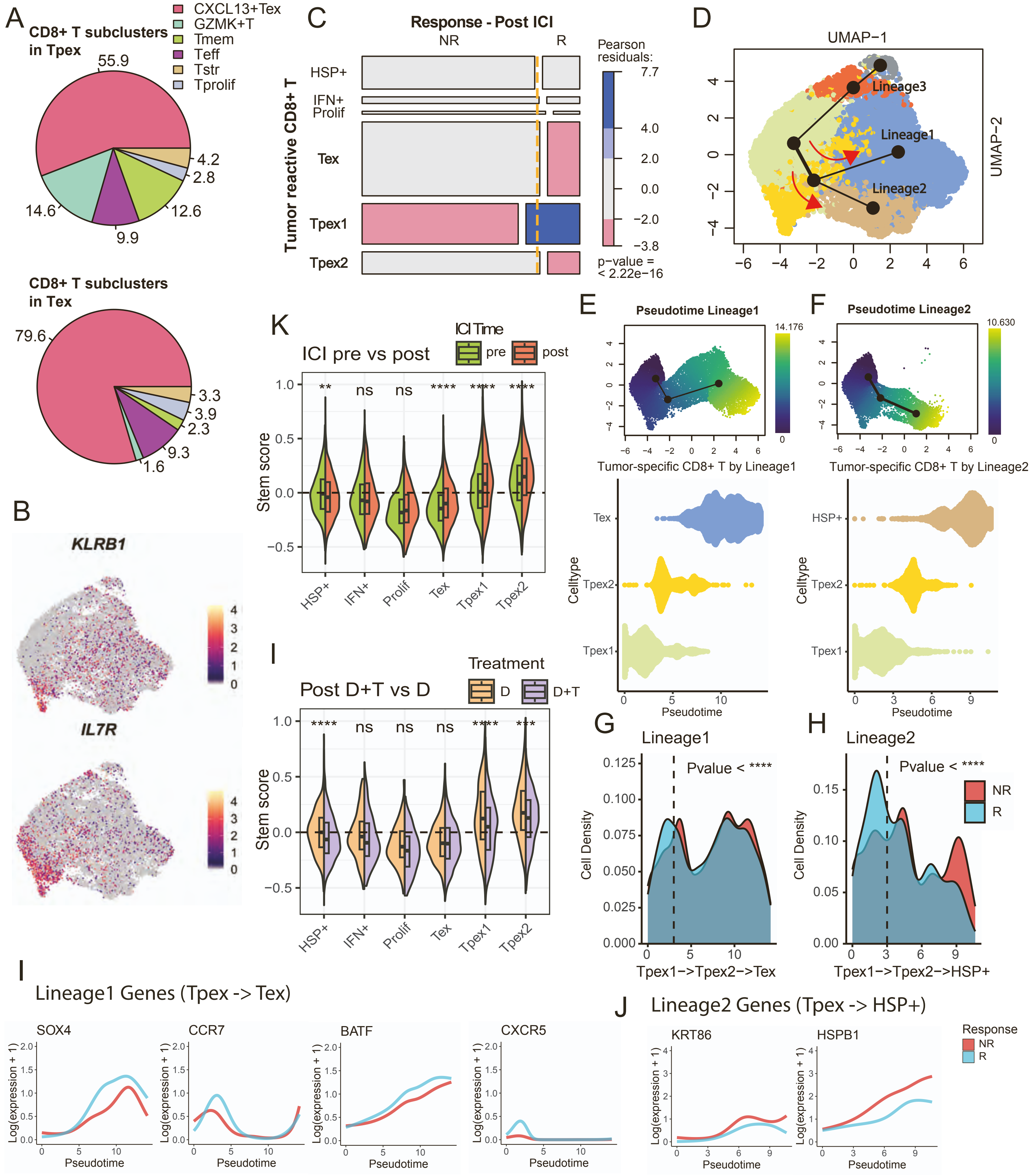

Figure S7 | Pseudotime analysis of tumor-specific CD8+ T cells. Related to Figure 5. A. Proportion of CD8+ T cell subclusters that were incorporated into Tpex (Tpex1 and Tpex2, left) and Tex (right). **B.** Feature plot of gene KLRB1 (top) and IL7R bottom for tumor-specific T cells UMAP dimensions. **C.** Mosaic plot of all tumor-specific CD8+ T cells divided by ICI response at post treatment. The yellow line indicates the expected ratio. Pearson residual P-values are colored red or blue if significantly depleted or enriched respectively. **D.** Identified lineages by slingshot overlayed in UMAP dimension of tumor-specific CD8+ T cells. Lineage 1 and 2 were considered for this study. **E-F.** Pseudotime value of determined lineage in the UMAP dimension (top) and the cell types modeled along the determined pseudotime (bottom). Lineage 1 is shown in **(E)** and Lineage 2 is shown in **(F)**. **G-H.** Density plot of cell divided by response group along pseudotime lineage 1 (left) and lineage2 (right) modeled via slingshot. P-values are calculated via Kolmogorov-Smirnov test. Dashed vertical line represents pseudotime value 3. **I-J.** Representative genes that showed statistically significant difference (P-value < 0.01) between response group along the modeled trajectory lineage1 **(I)** and lineage2 **(J)**, calculated by TradeSeq. **K.** Split violin plot of stem signature score from Sade-Feldman et al. in each tumor-specific CD8+ T cell subclus-ter divided by pre- or post-ICI. P-values are calculated by two-sided Wilcox rank sum test. **L.** Split violin plot of stemness signature gene set score for each cell by Sade-Feldman et al. in different treatment groups (D: monotherapy, D+T: combination therapy), grouped by tumor-specific CD8+ T subclusters.

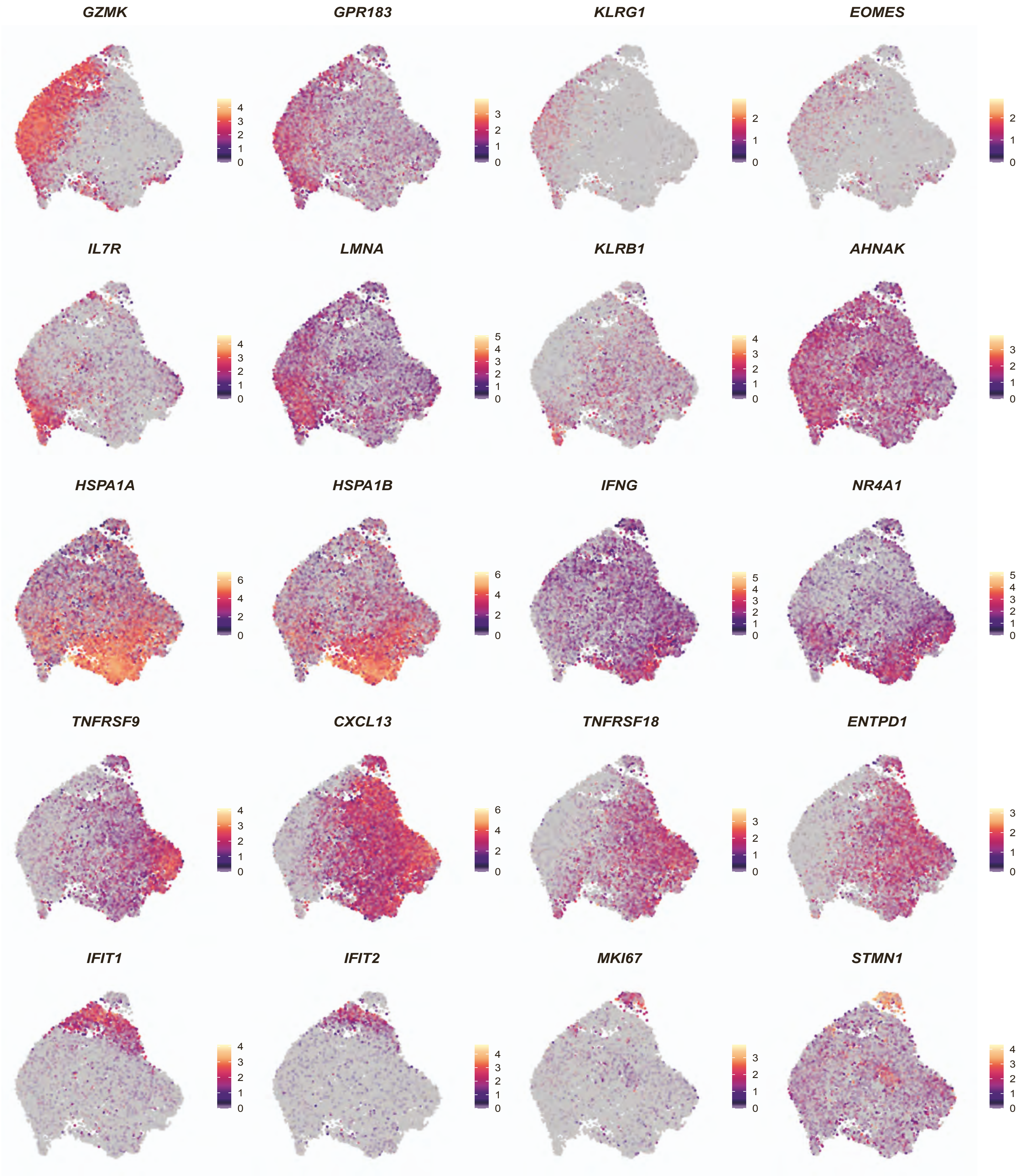

Figure S8 | Key gene expressions in tumor specific CD8+ T cells. Related to Figure 5. Feature plot of representative genes specifically upregulated in tumor specific CD8+ T cell subclusters

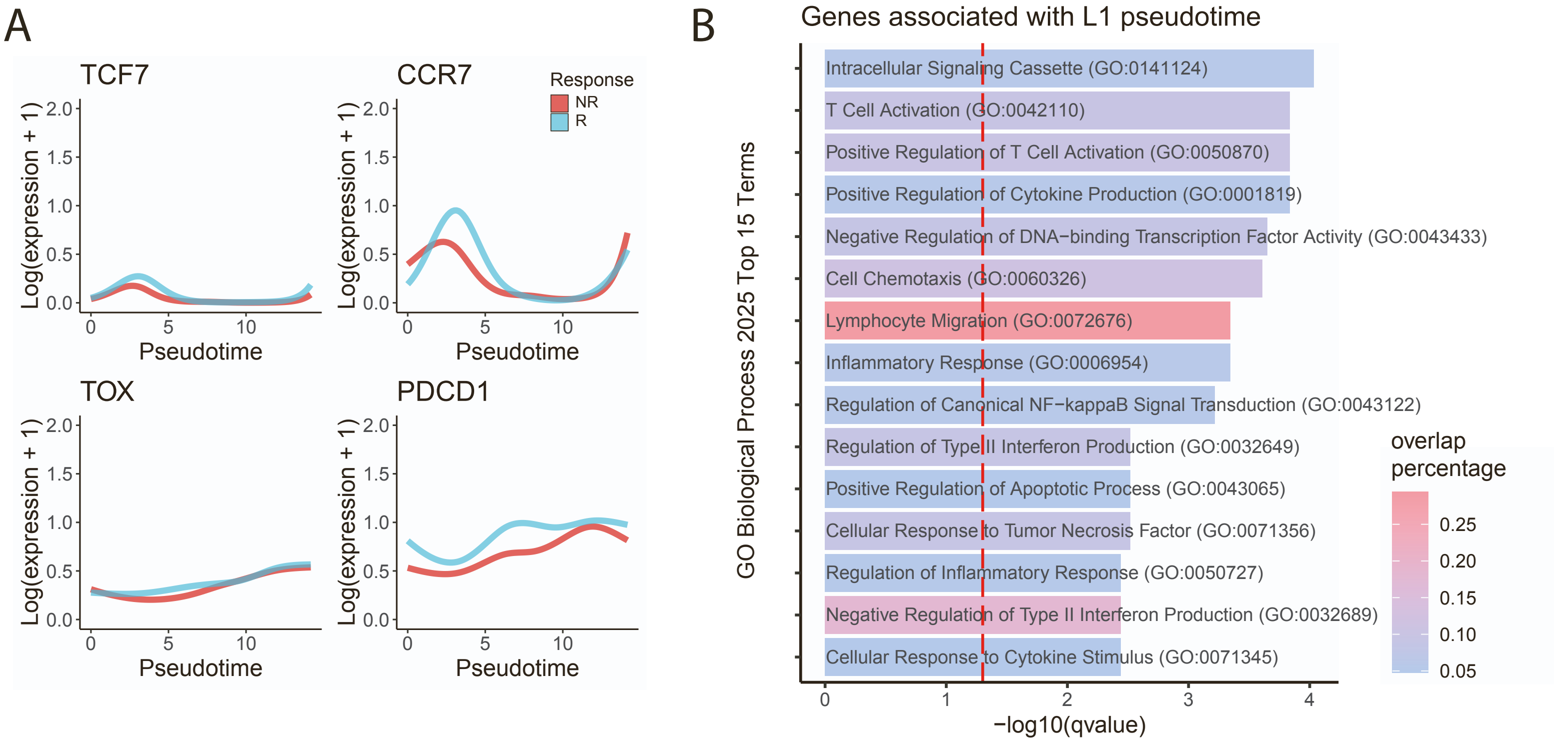

Figure S9 | Lineage analysis for gene dynamics and functions. Related to Figure 5. A. Expression dynamics of four representative genes across L1 pseudotime (Tpex1 to Tex) associated with T cell ICI response. **B.** The top 15 enriched Gene Ontology Biological Processes (2025 database) among all genes significantly associated with L1 pseudotime were identified using Fisher's exact test (adjusted P-value < 0.01, Benjamini–Hochberg correction).

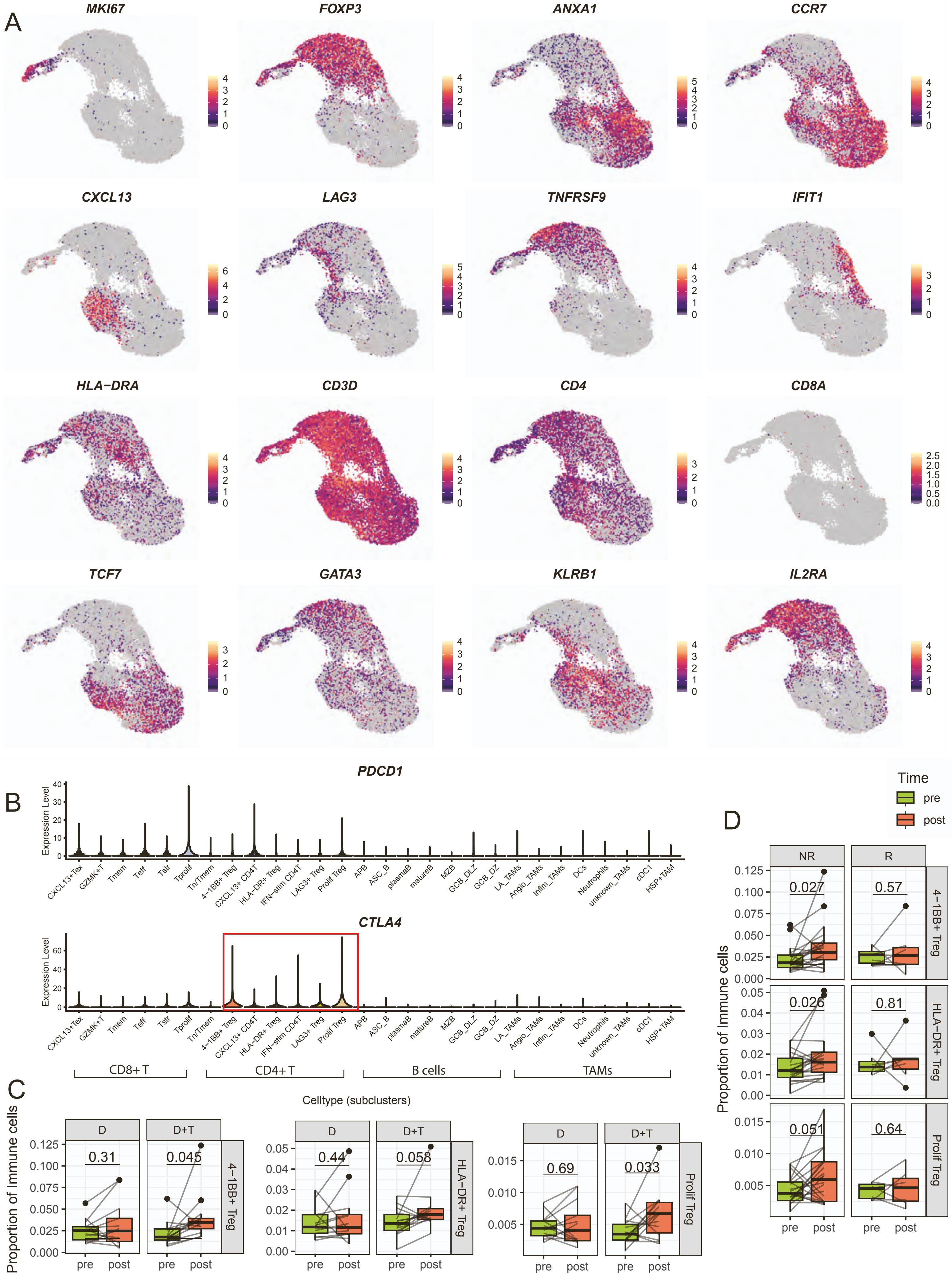

Figure S10 | CD4+ T cell gene expression patterns and proportional differences. Related to Figure 6. A. Feature plot Ωof representative genes specifically upregulated in tumor specific CD4+ T cell subclusters. **B.** Violin plot for PDCD1 and CTLA4 in all identified immune cell types within the dataset. **C.** Connected bar plot showing relevant CD4+ subsets proportions (as all immune cells as baseline) divided by treatment group and treatment timepoint. Paired t-test P-values denoted.

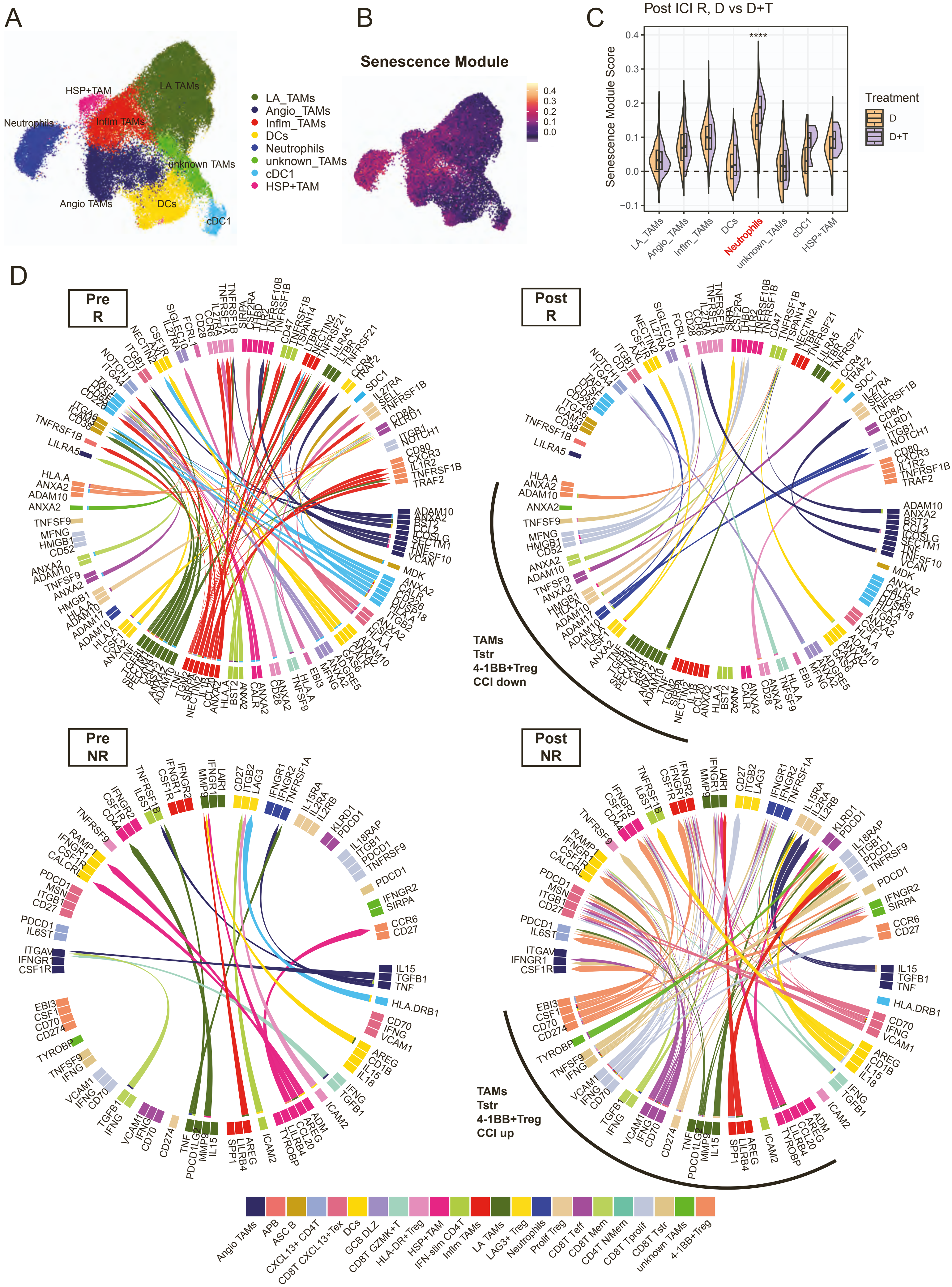

Figure S11 | Cell-cell interaction of top receptor and ligands across all cell types. Related to Figure 3 and Figure 6. A. UMAP of neutrophils and tumor associated macrophages (TAMs). **B.** Senescence module score colored in the UMAP dimen-sion. **C.** Violin plot of senescence module score divided by treatment group. P-values are calculated via Wilcoxon rank sum test. D. Top 50 ligand-receptor pairs that significantly increased interaction post therapy for each response group as measured by MultiNicheNet.
